# Vulnerability-Based Critical Neurons, Synapses, and Pathways in the *Caenorhabditis elegans* Connectome

**DOI:** 10.1371/journal.pcbi.1005084

**Published:** 2016-08-19

**Authors:** Seongkyun Kim, Hyoungkyu Kim, Jerald D. Kralik, Jaeseung Jeong

**Affiliations:** 1 Department of Bio and Brain Engineering, Korea Advanced Institute of Science and Technology (KAIST), Daejeon, Republic of Korea; 2 Program of Brain and Cognitive Engineering, College of Engineering, Korea Advanced Institute of Science and Technology (KAIST), Daejeon, Republic of Korea; George Mason University, UNITED STATES

## Abstract

Determining the fundamental architectural design of complex nervous systems will lead to significant medical and technological advances. Yet it remains unclear how nervous systems evolved highly efficient networks with near optimal sharing of pathways that yet produce multiple distinct behaviors to reach the organism’s goals. To determine this, the nematode roundworm *Caenorhabditis elegans* is an attractive model system. Progress has been made in delineating the behavioral circuits of the *C*. *elegans*, however, many details are unclear, including the specific functions of every neuron and synapse, as well as the extent the behavioral circuits are separate and parallel versus integrative and serial. Network analysis provides a normative approach to help specify the network design. We investigated the vulnerability of the *Caenorhabditis elegans* connectome by performing computational experiments that (a) “attacked” 279 individual neurons and 2,990 weighted synaptic connections (composed of 6,393 chemical synapses and 890 electrical junctions) and (b) quantified the effects of each removal on global network properties that influence information processing. The analysis identified 12 critical neurons and 29 critical synapses for establishing fundamental network properties. These critical constituents were found to be control elements—i.e., those with the most influence over multiple underlying pathways. Additionally, the critical synapses formed into circuit-level pathways. These emergent pathways provide evidence for (a) the importance of backward locomotion, avoidance behavior, and social feeding behavior to the organism; (b) the potential roles of specific neurons whose functions have been unclear; and (c) both parallel and serial design elements in the connectome—i.e., specific evidence for a mixed architectural design.

## Author Summary

One of the most important scientific aims is to uncover the functional design principles of nervous systems. To reach this aim, it is useful to examine a complex nervous system that is both well specified and highly tractable, making the nematode roundworm *Caenorhabditis elegans* an attractive model system, especially since it is the only complete connectome currently available for analysis. In this computational study, we tested the effects of individual attacks on every neuron and synaptic connection in the *C*. *elegans* connectome to identify and characterize the most critical constituents of the network by quantifying the changes in key network properties of the connectome that influence information processing. Our analysis identified 12 neurons and 29 synapses critical to clustering, information integration and propagation. These critical constituents formed circuit-level structures that control network processing in the *C*. *elegans* connectome. We believe our study provides a significant advance in the understanding of the network topology of the *C*. *elegans* connectome, and provides insights into the fundamental architectural design of complex nervous systems.

## Introduction

Understanding the fundamental architectural design of nervous systems will provide insights into nervous system function and how mental illness arises from its dysfunction. The design derives from a trade-off between physical wiring costs and functional complexity [1–4]. Nervous systems mediate behavior to achieve the organism’s goals, and thus the network must be composed of behavioral circuits (e.g., to forage, to avoid danger, to mate). The punitive nature of wiring costs, however, demands highly efficient solutions, producing short and shared pathways whenever possible. How then do nervous systems solve the problem of having distinct behavioral circuits, on the one hand, and near maximized sharing of pathways and information, on the other? To determine this, it is useful to examine a nervous system that is both well specified and highly tractable, making the nematode roundworm *Caenorhabditis elegans* an attractive model system, especially since its complete connectome is available for analysis.

Considerable progress has been made in delineating behavioral circuits [5–9] of the *C*. *elegans* including, for example, mechanosensation [10–12], chemosensation [13], feeding [14, 15], exploration [14, 15], egg laying [16–18], mating [7, 19–22], and tap withdrawal [23, 24] (i.e., avoidance of a vibrating “tap”). These circuits appear to follow a general pattern of sensory neurons (S) to 1–3 layers of interneurons (I), with the final interneuron layer being command interneurons (i.e., direct control of motor neurons), to one or more motor neurons (M) that directly control muscle activity (i.e., S → I → M pattern) [7, 14, 25]. Some subcircuits have also been characterized, for example, for both forward and backward locomotion, and more generally for foraging behavior [6, 14]. Nonetheless, many details remain unclear, not only with respect to the specific functions of every neuron and synapse, but also the extent to which the behavioral circuits are largely separate and parallel versus having a more integrative and serial design (i.e., input → central processing → output) [7, 14]. These issues are especially exacerbated in the *C*. *elegans* connectome because it is highly interconnected, with evidence that behavioral functions such as navigation entail activity across a large fraction of the nervous system [26].

Graph theoretic analysis of neuronal networks provides a normative approach to help quantitatively specify the network structure and dynamics—in which neurons are network *nodes*, and synapses (whether chemical or gap junctions) are *connections* or *edges* between the nodes. Important network properties of the connectome have been established, including a layout that is nearly optimized to minimize wiring costs [1, 3, 4, 8, 27]. The near optimal wiring suggests strong selection pressure on efficiency, and thus, near optimally efficient information processing: e.g., path sharing whenever possible. And characteristics have been identified that reflect evolved complexity in light of this constraint, especially small-worldness (i.e., high clustering for functional specialization, together with significant interconnections across the network for efficient information integration and transfer), and nonrandom distributions of highly connected neurons as well as those that support high volume traffic [4, 7, 8, 27–30]. Larger information processing structures in the network have also been identified, such as a set of highly connected interneurons that form hubs and “rich clubs” that integrate information to drive locomotion [27, 31]. Modularity analyses have also attempted to delineate the main functional modules based on high interconnectivity within modules versus lower across them [8, 27, 28]. Although specific results across studies differ based on the modularity algorithm used, these studies demonstrate that identified modules align with known behavioral circuits, providing some evidence for separable behavioral circuits. At the same time, the results also show elements of an integrative and serial design (i.e., to the extent modules align with input, central, and output processing layers). Taken together, current findings may suggest a fundamentally mixed architectural design.

An important next step, then, is to build on these findings by unequivocally identifying specific critical substructures of the connectome, which will in turn help clarify the architectural design principles. To this end, we take an evolutionary and developmental (*evo-devo*) perspective and seek to identify the most critical elements of this information processing system, i.e., those whose addition provided the most ‘bang for the buck’ for information propagation, integration and processing [32–34]. To determine this, in the current study we conducted a computational network-vulnerability analysis that systematically removed network components and calculated the effects of each loss on key network properties, with the view that functional loss upon removal reveals what their addition enables. We also take the perspective that critical components in real-world nervous systems may not always depend on local individual properties independent of context, such that significance may emerge from this context. The vulnerability analysis also enables an assessment of these contextual effects [35, 36]. Using this approach, we tested all neurons and synapses to assure no misassignments: i.e., some considered critical when not (e.g., highly connected but redundant), or those that may appear not, but in fact are, from a more global network perspective.

## Results

### Identifying critical neurons

We analyzed the most recent published connectome of the *C*. *elegans* hermaphrodite as a combined (i.e., chemical synapses and gap junctions), directed, and weighted network (see Methods and S1 Text) [5, 15, 29]. The S1 Text contains an examination of the general network properties of the intact network, including circular wiring diagrams, comparisons of the three neuron classes (sensory, interneurons, and motor) on different network properties, and robustness and information propagation analyses. We then sought to identify the most critical neurons in the *C*. *elegans* connectome. To do this, we asked which ones had the greatest impact on network function when they are lost. Thus, we conducted a vulnerability analysis, attacking each of the 279 neurons in the connectome and calculating the change in global network properties when the neuron is removed (see Methods for analysis details and explanation of notation used; S1 File) [35, 36]. Three fundamental properties that determine efficient information processing in a network are (1) specialized processing in the form of local clusters, (2) global information integration and coordination via short path lengths between nodes, and (3) the control of information flow by providing a route that is shared by multiple pathways. These three properties were assessed by calculating the network vulnerability, *V*, (i.e., the relative change in value after elimination of the network component) with respect to the clustering coefficient (*V*_*C*_), global efficiency (*V*_*E*_), which assesses the network’s average path length, and the average betweenness centrality (*V*_*B*_), which represents the average of number of shortest paths that travel through each network component (i.e., neurons here, synapses below) (detailed in the Methods and S1 Text).

**Fig 1:**
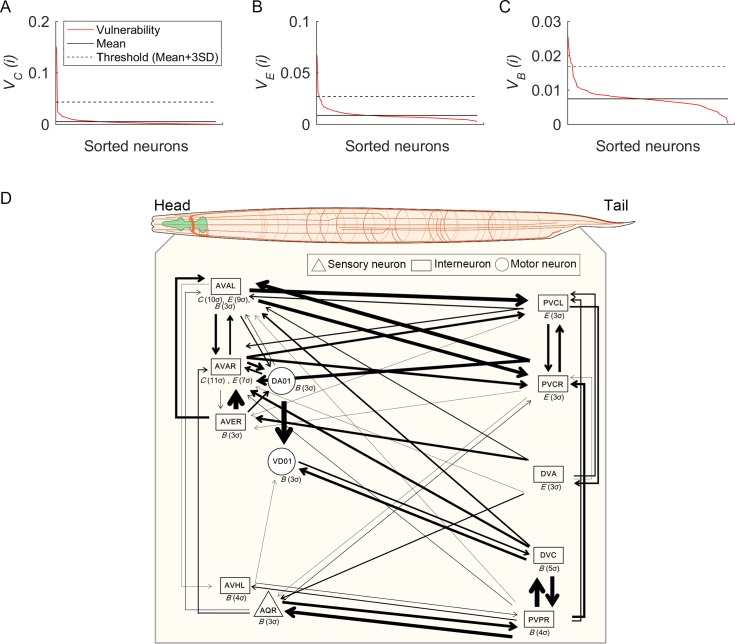
Vulnerability results for each network property and a visualization of critical neurons. (A-C) Sorted vulnerability values (red) for each network property (Clustering: *C*, Efficiency: *E*, and Betweenness: *B*) induced by 279 single neuronal attacks. Mean vulnerability value for 279 neurons are indicated by the solid black lines; black dashed lines indicate the threshold value (mean plus 3SDs). (D) Depiction of the 12 critical neurons with their connections to each other along the anterior-posterior body axis in the *C*. *elegans* body context. The network property for each neuron is shown (*C*, *E*, and *B*) with the critical grade in parenthesis; thickness of links indicates the weight grade of a link.

To determine the critical neurons, we reasoned that the most influential ones should yield network vulnerability scores after deletion that are clear outliers from the rest of the population, reflecting extreme functional loss in the network with respect to the specific fundamental network property. The most conservative criterion used in the Towlson et al. [27] study to identify the *rich club* was selecting those that were 3SD or more above the average, which we adopted here (Fig 1; S2 File). This stricter criterion enabled the identification of the most critical neurons, it provided a standard value often used in identifying outliers (3SD), and it continued the convention established by Towlson et al. [27], enabling a direct comparison of findings across studies. Thus, we defined a neuron as critical for a given network property when a deletion of the neuron produced a vulnerability over three SDs above the mean vulnerability value of all 279 neuronal attacks.

Our analysis identified 2, 5, and 8 critical neurons for *V*_*C*_, *V*_*E*_, and *V*_*B*_, respectively, yielding 12 (i.e., 4.3%) critical neurons in all (Table 1; S1 and S2 Files). Of the 12 critical neurons, 9 are interneurons, 2 are motor neurons, and 1 is sensory, reflecting the general importance of interneurons in information processing in the *C*. *elegans* connectome, as has been reported previously [27]. Moreover, of the 7 interneurons with known function, 5 are considered command interneurons that control locomotion [27].

**Table 1:** The list of 12 critical neurons.

Neuron	*V*_*C*_	*V*_*E*_	*V*_*B*_	Functional role [5]	Birth Time (min)
AQR (S)			3σ	Aerotaxis (O_2_ and CO_2_), regulating social feeding and bordering behavior (i.e., aggregation in densest part of bacterial lawn), and suppressing innate immunity	1280
AVAL (I)	10σ	9σ	3σ	Backward locomotion	312
AVAR (I)	11σ	7σ		Backward locomotion	311
AVER (I)			3σ	Backward locomotion	329
AVHL (I)			4σ	Unknown yet	347
DA01 (M)			3σ	Backward locomotion	309
DVA (I)		3σ		Mechanosensory integration; Sensory-motor integration during locomotion	296
DVC (I)			5σ	Backward locomotion	351
PVCL (I)		3σ		Forward locomotion modulating response to harsh touch to tail	449
PVCR (I)		3σ		Forward locomotion modulating response to harsh touch to tail	450
PVPR (I)			4σ	Ventral cord pioneering (first during development for axon guidance)	310
VD01 (M)			3σ	Sinusoidal body movement	1460

S: Sensory neuron, I: Interneuron, M: Motor neuron. *V_C_, V_E_, V_B_*: Vulnerability scores for network clustering, efficiency, and betweenness.

### Inner-layer critical neurons develop early, peripheral ones late

**Fig 2:**
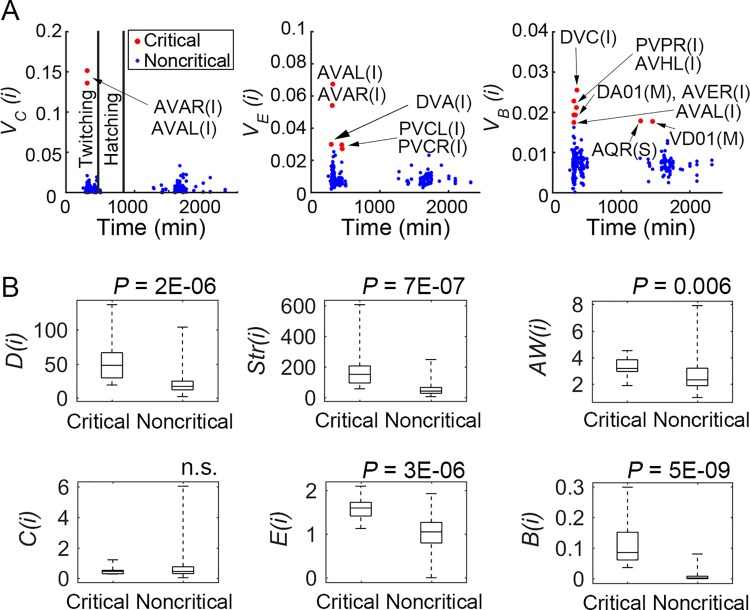
The relation of vulnerability scores to neuronal birth times and intact network properties of the critical neurons compared with the rest of the nervous system. (A) Critical neurons (which produced changes in vulnerability measures larger than 3SDs) are indicated for each network property (Clustering: *C*, Efficiency: *E*, and Betweenness: *B*) by the red dots and their names. Blue dots indicate the rest of the nervous system. Most critical neurons are born in the early or intermediate phases. (B) The distributions (first, second, i.e., median, and third quartiles, and minimum to maximum) and statistical comparisons of critical and noncritical components for each network property (Degree: *D*, Strength: *Str*, Average Weight: *AW*, *C*, *E*, and *B*). The critical group has significantly higher values than noncritical neurons except for *C*.

To obtain further insight into the critical neurons, we examined their development times (Fig 2A; Table 1) [27]. All critical neurons for both the *V*_*C*_ (2 interneurons) and *V*_*E*_ (5 interneurons) analyses developed early. For *V*_*B*_, 5 interneurons and one motor neuron (DA01) developed early, whereas 2 neurons developed late (after hatching): one sensory (AQR) and one motor (VD01). Taken together, all interneurons and 1 motor neuron (DA01) developed early, whereas, one sensory and 1 motor neuron developed late. Thus, as found by Towlson et al. [27], we also found that all of the critical interneurons formed in the early development phase before twitching (470 min after fertilization; first visible motor activity), presumably attesting to their importance in establishing the connectome topology. In addition, we note that the early developing motor neuron (DA01) is less peripheral than the late developing one with respect to functional connectivity (VD01) in the fully formed adult connectome [6]. Thus, our results also suggest a general ‘inside-out’ developmental pattern, with the most peripheral neurons tending to develop last, presumably requiring environmental experience after hatching [27, 37].

### Neuron criticality results for *V*_*C*_, *V*_*E*_, *and V*_*B*_ and their relationship to biological function

Examining the specific results for *V*_*C*,*E*,*B*_, the AVA neurons stand out, being critical for multiple network properties (Table 1; S1 File). AVAL and AVAR were critical for both *V*_*C*_ and *V*_*E*_, meaning that they most affect network clustering and efficiency. AVAL was additionally critical for *V*_*B*_, although this measure (i.e., *V*_*B*_(*i*)) actually decreased when AVAL was removed (without taking the absolute value) (S1 File). This decrease indicates that a main pathway segment was removed with AVAL, leaving longer alternative routes (resulting in increased *B*(*i*) overall, *X*_*B*_(*i*)). Thus, the results show that the AVA neurons (and AVAL in particular) are dominant ones in the connectome, critically influencing network clustering, interconnectivity, and information flow. Given AVA’s known role in backward movement control, these results suggest that backward movement likely plays a key role in locomotor maneuverability in potentially multiple biological functions; and to the extent backward movement in response to environmental stimuli might be construed as avoidance behavior, the results might also highlight the significance of avoidance behavior in general. We discuss possible biological function further below.

For *V*_*E*_, 5 neurons were found to have the greatest effect on global efficiency: besides the two AVA neurons, DVA (I) and PVCL/R (I) (i.e., both left and right) were also identified (Table 1; S2 File). All five critical neurons for *V*_*E*_ are interneurons, which would indeed be those expected to have significant influence on global efficiency (yet only these 5 were found to be critical). Since all have been implicated in either forward locomotion for avoiding aversive stimuli or backward locomotion, it suggests that the most influential nodes on global efficiency in the connectome perform locomotion control, and again, especially with respect to backward locomotion and avoidance behavior.

For *V*_*B*_, 8 neurons were found to have the greatest effect on betweenness centrality: 1 sensory (AQR), 5 interneurons (AVAL, AVER, AVHL, DVC, PVPR), and 2 motor (DA01, VD01) (Table 1; S1 File). Thus, with respect to network information propagation, all neuronal types (S, I, and M), related to potentially multiple biological functions are implicated, which we examine further in the synapse analysis.

During this study, AVHL and DVC were conspicuous critical neurons whose biological function had been unknown (there has since been new evidence for DVC involvement in locomotion) [38]. We also note that due to the integrative nature of the *C*. *elegans* connectome, it is possible that many neurons (especially interneurons) participate in multiple functions. We therefore conducted an analysis based on known biological function of neighbors to help determine the possible functions of AVHL and DVC (S1 Text; S8 Fig). Indeed, our analysis suggests multiple possible functions for both neurons, most notably ventral cord pioneering, chemotaxis, and locomotion for AVHL, and locomotion and ventral cord pioneering for DVC. We discuss possible biological functions for these neurons further below. We also note that although ventral cord pioneering is important for axon guidance during neuronal development of the *C*. *elegans* connectome, if these neurons remain integrated in the connectome in the fully developed adult, they likely participate in functions other than ventral cord pioneering. This consideration would then point to chemotaxis and locomotion for AVHL, and locomotion for DVC in the adult neuronal network.

### Critical neurons are network control structures

We next attempted to better understand the general characteristics of the critical neurons. For example, were they the most highly connected? Degree does appear important, with 6 critical neurons being in the top 10 for degree (these 6 being members of the rich club [27]). However, we also found 6 other critical neurons, and none were among the next 18 for degree, with all 12 critical neurons being only in the top 113 (of 279 total) (Tables D and E in S1 Text). To further examine the properties of the critical neurons, we first compared all critical neurons to the noncritical ones on multiple network factors using the Mann-Whitney U test (Fig 2B). The critical neurons have higher degree (*D*), strength (*Str*), average weight (*AW*), nodal efficiency (*E*(*i*)), and nodal betweenness centrality (*B*(*i*)) values than the noncritical neurons. Only the nodal clustering coefficient (*C*(*i*)) showed no differences between the critical and noncritical groups. Thus, as a group, the critical neurons were more connected, more strongly connected, more closely connected to others in the network (i.e., the shortest average path lengths), and generally positioned as important *control centers* regarding the number of shortest pathways passing through them and thus the number of pathways influenced by them. These results support others that have shown the significance of nodal network properties such as degree and betweenness centrality in the connectome [7, 8, 27–29].

Overall, multiple factors distinguish the critical neurons, but it is important to clarify their impact on each vulnerability measure *V*_*C*,*E*,*B*_ individually. One might assume that each vulnerability measure would be most affected by its corresponding nodal property: e.g., individual neuron values for clustering coefficient (*C*(*i*)) determining global *V*_*C*_. However, this is not what we found—in fact in no case was the same local nodal property the leading factor influencing global network vulnerability. To evaluate the relationship of global *V*_*C*,*E*,*B*_ to nodal network properties of the intact network, we examined correlations of *D*, *Str*, *AW*, *C*(*i*), *E*(*i*), *B*(*i*) to *V*_*C*,*E*,*B*_ without taking the absolute values (Table F in S1 Text). The S1 Text contains a detailed discussion of the results. In sum, neurons most critical for network clustering (*V*_*C*_) send dual (or more) projections to other neurons that project to each other, producing the largest clusters. Those most critical for global efficiency (*V*_*E*_) generally have the most influence—or *control*—in the network, regarding the number of pathways sharing them and thus the number influenced by them. Thus, the critical *V*_*E*_ neurons do not necessarily have high individual efficiency values, but they do normally have high individual betweenness values. And those critical for network betweenness (*V*_*B*_) also appeared to be those with most control in the network, but the effect is more contextual—it depends on the other possible available routes when the node is lost (detours), the loss in nodal betweenness centrality of the attacked node, and the changes generated in others due to the loss of the attacked connections. Most critical neurons for *V*_*B*_ also tended to be connected to other nodes with high nodal betweenness centrality, creating a linked control structure.

### Identifying critical synapses

**Fig 3:**
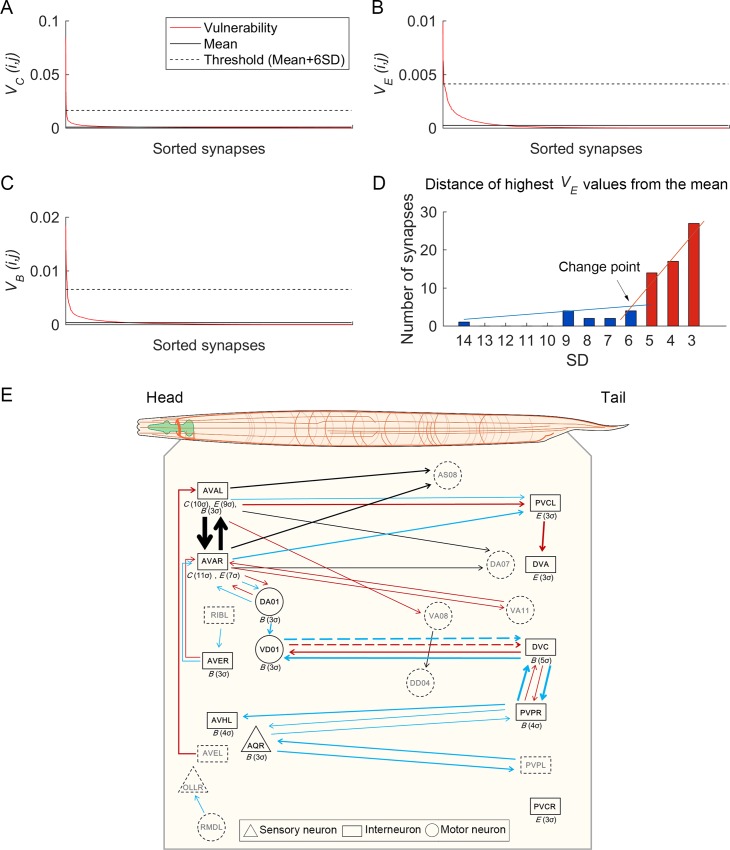
Vulnerability results for each network property and a visualization of critical synapses. (A-C) Sorted vulnerability values (red) for each network property (Clustering: *C*, Efficiency: *E*, and Betweenness: *B*) induced by all 2990 single synaptic attacks. Mean vulnerability values for 2990 synapses are indicated by the solid black lines; black dashed lines indicate the threshold value (mean plus 6SDs). (D) A depiction of the procedure used to determine the criterion for selecting critical synapses. The histogram of the distances of the highest *V*_*E*_(*i*,*j*) values from the mean is shown. The intersection of the linear fitting of the blue bars (>6SD) and the linear fitting of the red bars (<6SD) reveals a change point at 6SD (y-intercepts were changed for clearer view). Because 6SD was the highest change point value among the three network properties, and thus the most conservative value, it was used as the criterion to identify critical synapses. (E) Depiction of the 12 critical neurons and 29 synapses along the anterior-posterior body axis in the *C*. *elegans* body context. The network property for each neuron is shown with the critical grade of a neuron in parenthesis; thickness of links indicates the critical grade of a link. For synapses, colors of links indicate network properties whose vulnerability value were over the threshold (6SDs) (black: *C*, red: *E*, and blue: *B*); if there were more than two critical measures for a synapse, each critical measure for the synapse was indicated with a separate colored link. Dashed shape indicates that the neuron is not critical but is connected with a critical neuron or critical synapse. For synapses, dashed lines indicate inhibitory synaptic connections (all others are excitatory).

The previous analysis of critical neurons determined the significance of each neuron’s constellation of connections to the network. An individual synapse analysis provides a finer grain analysis of each constituent element [39, 40]. We therefore next conducted a vulnerability edge analysis by attacking each one of the 2,990 synaptic connections (S3 File). Because the criterion used to identify critical neurons (3SD or above) proved too liberal for the synapses (potentially labeling too many as most critical and obscuring differences among them), we used the following procedure. First, for each list of *V*_*C*_, *V*_*E*_, and *V*_*B*_ values (Fig 3A–3C), we transformed the values to the distance from the mean in SD integer units (i.e., 1SD, 2SD, etc.), and ordered this list from highest to lowest. Second, we created a histogram distribution for each ordered list and identified the first major change in the slope (see Fig 3D). Third, we compared the SD values of the first change points for *V*_*C*_, *V*_*E*_, and *V*_*B*_ and used the most conservative value (the highest SD distance) as the criterion for all three vulnerability measures. From this procedure, 6SD was identified from the *V*_*E*_ values and used as the criterion to identify the critical synapses (Fig 3A–3D).

The analysis identified 7, 13, and 17 synapses as critical for *V*_*C*_, *V*_*E*_, and *V*_*B*,_ respectively, implicating 29 (i.e., ~1%) critical synapses (Table 2; Fig 3). 28 of the 29 synapses are excitatory, suggesting that they promote information transmission. The one exception, VD01 (M) → DVC (I), is inhibitory, which we note further below. In addition, 26 of the 29 pairs (89.7%) contain at least one interneuron, 14 (48.3%) contain at least one motor neuron, and 5 (17.2%) contained one sensory neuron. Thus, the critical synapses also appear to relate predominately to interneurons, but also included motor and sensory neurons.

**Table 2:** The list of 29 critical synapses.

Synapse			*V*_*C*_	*V*_*E*_	*V*_*B*_	Functional role [5]
1. AQR (S)	→	PVPL (I)			9σ	Aerotaxis, social feeding, bordering, immunity responses → Ventral cord pioneering
2. AQR (S)	→	PVPR (I)			7σ	Aerotaxis, social feeding, bordering, immunity responses → Ventral cord pioneering
3. AVAL (I)	→	AS08 (M)	12σ			Backward locomotion → Locomotion
4. AVAL (I)	→	AVAR (I)	32σ			Backward locomotion → Backward locomotion
5. AVAL (I)	→	DA07 (M)	7σ			Backward locomotion → Backward locomotion
6. AVAL (I)	→	PVCL (I)		9σ	6σ	Backward locomotion → Forward locomotion
7. AVAL (I)	→	VA08 (M)		6σ		Backward locomotion → Locomotion
8. AVAR (I)	→	AS08 (M)	12σ			Backward locomotion → Locomotion
9. AVAR (I)	→	AVAL (I)	28σ			Backward locomotion → Backward locomotion
10. AVAR (I)	→	DA01 (M)		6σ	8σ	Backward locomotion → Backward locomotion
11. AVAR (I)	→	DA07 (M)	7σ			Backward locomotion → Backward locomotion
12. AVAR (I)	→	PVCL (I)			10σ	Backward locomotion → Forward locomotion
13. AVAR (I)	→	VA11 (M)		8σ		Backward locomotion → Locomotion
14. AVEL (I)	→	AVAL (I)		9σ		Backward locomotion → Backward locomotion
15. AVER (I)	→	AVAR (I)		8σ	7σ	Backward locomotion → Backward locomotion
16. DA01 (M)	→	AVAR (I)		6σ	8σ	Backward locomotion → Backward locomotion
17. DA01 (M)	→	VD01 (M)			11σ	Backward locomotion → Sinusoidal body movement
18. DVC (I)	→	PVPR (I)		7σ	17σ	Backward locomotion → Ventral cord pioneering
19. DVC (I)	→	VD01 (M)		9σ	13σ	Backward locomotion → Sinusoidal body movement
20. PVCL (I)	→	DVA (I)		14σ		Forward locomotion → Mechanosensory integration
21. PVPL (I)	→	AQR (S)			9σ	Ventral cord pioneering → Aerotaxis, social feeding, bordering, immunity responses
22. PVPR (I)	→	AQR (S)			7σ	Ventral cord pioneering → Aerotaxis, social feeding, bordering, immunity responses
23. PVPR (I)	→	AVHL (I)			10σ	Ventral cord pioneering → Unknown
24. PVPR (I)	→	DVC (I)		7σ	17σ	Ventral cord pioneering → Backward locomotion
25. RIBL (I)	→	AVER (I)			6σ	Information integration → Backward locomotion
26. RMDL (M)	→	OLLR (S)			7σ	Foraging movements and head-withdrawal reflex → Mechanosensation and negative pathogen avoidance regulation
27. VA08 (M)	→	DD04 (M)	6σ			Locomotion → Sinusoidal body movement
28. VA11 (M)	→	AVAR (I)		6σ		Locomotion → Backward locomotion
29. VD01 (M)	→	DVC (I)		9σ	13σ	Sinusoidal body movement → Backward locomotion

S: Sensory neuron, I: Interneuron, M: Motor neuron. *V_C_*, *V_E_*, *V_B_*: Vulnerability scores for network clustering, efficiency, and betweenness.

Since inherently costly long-range connections should be minimized, one might expect an overrepresentation of these among the most critical synapses, as they would provide critical waypoints for the dispersed nervous system. Indeed, we did find a bias for long-range connections as calculated by the direct Euclidean distance of soma positions between two neurons: 16 of the 29 (55.2%), compared to roughly 10% in the entire connectome (Fig 3E) [1, 3, 4]. These connections were similar for all vulnerability results: for *V*_*C*_, 4/7 (57%); *V*_*E*_, 6/13 (46%); *V*_*B*_, 9/17 (53%).

Finally, regarding connectome laterality, we did find asymmetries between the left and right neuronal pairs, in which certain neuron types were critical only for the left or right (e.g., neurons AVER, AVHL, PVPR). Overall, however, we found that there is no clear overall laterality bias in the critical neurons (4 left, 5 right) or synapses (source node: 10 left, 12 right; sink node: 6 left, 8 right). The left/right differences that we did find, however, warrant future examination of laterality in the *C*. *elegans* connectome [41].

### The critical synapses form circuit-level structures related to biological function

We next examined the vulnerability edge results for each network property *V*_*C*,*E*,*B*_ more closely (Table 2). For *V*_*C*_, of 7 total, 2 were AVA connections to each other (i.e., AVAL (I) ←→ AVAR (I)), 4 were AVAL/R (i.e., both left and right) to motor neurons DA07 (M) and AS08 (M), and 1 was motor to motor (VA08 (M) → DD04 (M)). With respect to biological function, AVA, DAn, and VAn have well-established involvement in backward locomotor control, and DDn and ASn in the coordination of locomotion (between forward and backward). Thus, the results show that the most dominant synapses that drive coordinated local processing occurs with locomotor control, and in particular, backward locomotion.

The analysis of *V*_*B*_ identifies 17 critical synapses with the largest influence on betweenness centrality: 15/17 (88%) contain at least one interneuron in the pair, 6/17 (35%) at least one motor neuron, and 3/17 (18%) have one sensory neuron, with 7 of the 17 (41%) links being interneuron to interneuron (I → I), and the remaining 10 being some combination of sensory, interneurons, and motor neurons.

**Fig 4:**
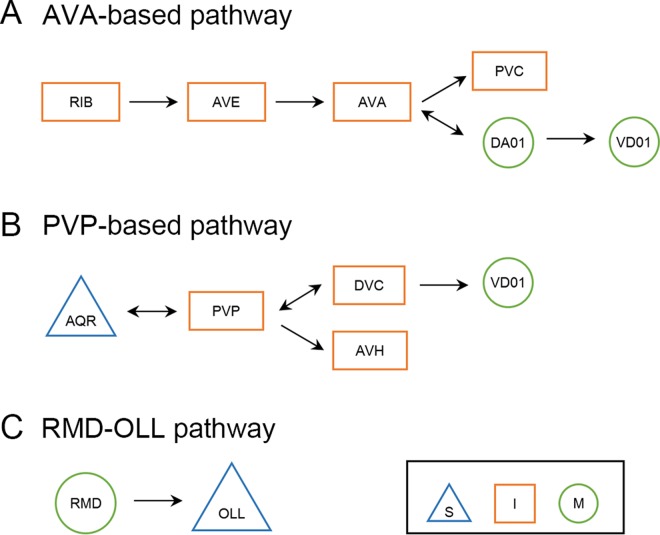
Three main critical synapse pathways as determined by the Vulnerability analysis for average betweenness (*V*_*B*_). Three main pathways for *V*_*B*_: the AVA-based, the PVP-based, and the RMD (M) → OLL (S) pathways (S: Sensory neuron, I: Interneuron, M: Motor neuron). These three main pathways emerged from the critical synapse analysis and well capture the converged findings from both the critical neurons and synapse vulnerability analyses. (A) The first AVA-based pathway begins with RIB (I) → AVE (I) → AVA (I) and has two routes after this: (a) AVA (I) ←→ DA01 (M) → VD01 (M) (ending at VD01 since VD01 → DVC (I) is inhibitory); and (b) AVA (I) → PVC (I). For route (b) (RIB (I) to PVC (I)), we found that average network betweenness centrality (i.e., *X*_*B*_(*i*,*j*)) actually increased with the AVAL/R (I) → PVCL (I) synapse removals (*V*_*B*_(*i*,*j*) had negative values without taking the absolute values). This suggests that the loss of the AVA → PVC connection leaves greater differences among the remaining alternative routes to PVC, perhaps leaving the well-established forward locomotion circuit as the main throughway (i.e., AVB (I) → PVC (I)). (B) The second PVP-based pathway, begins with AQR (S) ←→ PVP (I) and has two routes after this: (a) AQR (S) ←→ PVP (I) ←→ DVC (I) → VD01 (M); and (b) AQR (S) ←→ PVP (I) → AVH (I). We also note that the inhibitory projection of VD01 (M) → DVC (I) suggests a separation of the AVA- and PVP-based pathways and a competitive relationship between them. (C) The RMD (M) → OLL (S) link was also identified as a critical pathway segment.

To better understand the significance of these critical segments in the network for overall betweenness centrality, we examined their distribution. If these segments were independent, they should be fairly evenly distributed throughout the connectome. If, however, there were an interdependency among them, some pattern may emerge. In fact, we found that the synapses formed three separate pathways (see blue lines in Fig 3E). To see these more clearly we collapsed left and right neuron pairs (e.g., AVAL or AVAR as AVA). Justification for the left/right collapsing is taken from the biological work on the *C*. *elegans*, which normally shows symmetric function for neuron pairs [3, 6]. As shown in Fig 4A, the first *AVA-based critical pathway* begins with RIB (I) → AVE (I) → AVA (I) and has two routes after this. Route (a) (from RIB (I) to VD01 (M)) is in fact a significant segment of the well-established circuit for backward locomotion. Although the role of route (b) (RIB to PVC) has been suggested to be secondary coordination of backward with forward locomotion, the *V*_*B*_ analysis nonetheless suggests that this is an important pathway for locomotor control.

As shown in Fig 4B, the second *PVP-based critical pathway* begins with AQR (S) ←→ PVP (I) and has two routes after this. For route (a), the results suggest a critical sensory (AQR) to motor (VD01) circuit. To date, AQR has been implicated in aerotaxis (O_2_ and CO_2_), regulating social feeding (i.e., behavior when individuals aggregate at bacterial patches) and bordering behavior (i.e., aggregation in densest part of bacterial patch), and suppressing innate immunity [5, 42–44]. Since evidence shows that aerotaxis also regulates social feeding [43], we summarize these functions as the regulation of (1) social feeding [42, 43] and (2) internal immunity responses [44]. The motor neuron VD01 is implicated in locomotion [5, 6, 15]. Given that route (a) ends in locomotor behavior, the results suggest that the identified circuit is involved in social feeding. Moreover, the results thus predict involvement of both PVP and DVC in this behavioral circuit, whose functional roles in the fully developed adult network to date remain unclear (although there is evidence for PVP involvement in ventral cord pioneering during development; and there is new evidence for DVC involvement in locomotion) [38, 45–47]. More specifically, if we use the general behavioral circuit structure as a guide, S --> I_1_ --> I_2_ --> M, it provides further suggestive evidence for the functions of PVP (I_1_: sensorimotor integration) and DVC (I_2_: information integration, motor control)[7, 14]. The behavioral function of route (b) (AQR (S) ←→ PVP (I) → AVH (I)) is less clear. However, since route (a) implicates PVP in social feeding behavior, this may also implicate route (b) in the same, suggesting a possible functional role for AVH, whose function currently remains unknown. The more local analysis of the potential biological functions of AVH and DVC based on the functions of their neighbors (reported in the S1 Text) also appears to further corroborate these findings by implicating both AVHL and DVC in locomotor control (among other possible functions, which is also to be expected).

Finally, the *V*_*B*_ analysis also identified the RMD (M) → OLL (S) link as a critical pathway segment (Fig 4C). OLL has been implicated in aversive stimulus sensation and RMD in avoidance responses of the nose/head. Interestingly, the motor to sensory link as an important pathway highlights the loop structure in the *C*. *elegans* circuitry, with the motor to sensory link closing the loop [7]. This loop structure component suggests that multiple motor signals likely converge on sensory processing, suggesting significant action-influenced perception. Indeed, given the bidirectional links in the two main pathways between motor and interneurons, interneurons among themselves, and interneurons and sensory neurons, the *V*_*B*_ results suggest that these are important recurrent connections in the connectome circuitry, and further suggest that recurrent feedback loops for central processing (middle layers) and action influences on perception are important general principles of nervous system function [7].

**Fig 5:**
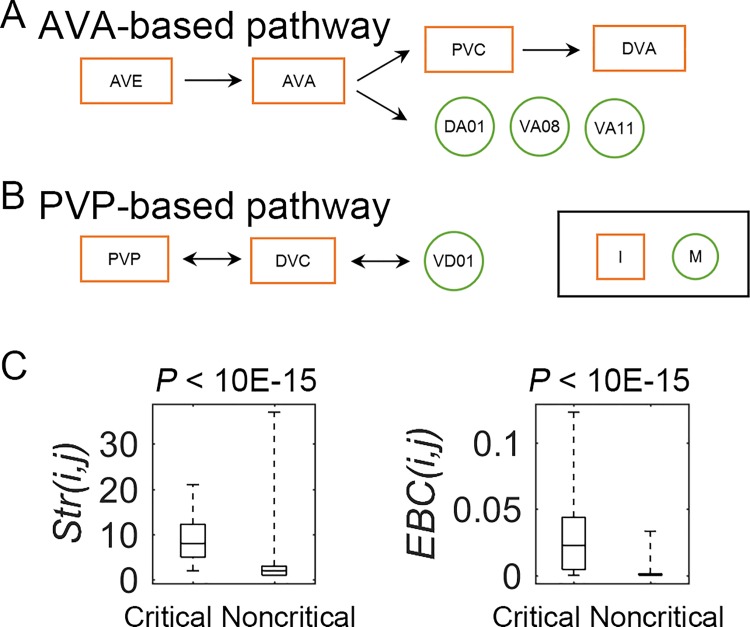
Two critical pathways identified by the Vulnerability analysis for global efficiency (*V*_*E*_), and intact network properties of the critical synapses compared with the rest of the nervous system. (A) The first critical pathway is also AVA-based, and starts with the AVE (I) → AVA (I) segment (S: Sensory neuron, I: Interneuron, and M: Motor neuron). Then there are four routes. The first three routes run from AVA to three different motor neurons: DA01 (same as *V*_*B*_), VA08, and VA11. As with AVE and AVA, both DAn and VAn (dorsal and ventral) neurons participate in backward locomotor control. The fourth route is AVE (I) → AVA (I) → PVC (I) → DVA (I), with the latter link actually being the strongest with respect to SD size (14SDs). As was found for *V*_*B*_, the *V*_*E*_ analysis shows the significance of the AVE (I) → AVA (I) → PVC (I) connection, and provides additional evidence for a relationship to DVA. DVA has been implicated in sensorimotor integration during locomotion, normally for avoidance behavior, and again suggests coordination of forward and backward locomotion (given its relationship to AVA). (B) The second critical pathway is PVP (I) ←→ DVC (I) ←→ VD01 (M), which was also identified by *V*_*B*_ as a component of the PVP-based pathway, and thus provides further evidence for a PVP, DVC, and VD01 relationship, and the importance of locomotor control. (C) The distributions (first, second, i.e., median, and third quartiles, and minimum to maximum) and statistical comparisons of critical and noncritical synapses for each network measure (Strength: *Str* and Edge Betweenness Centrality: *EBC*). The critical group has significantly higher *Str* and *EBC* values than the noncritical group.

The *V*_*E*_ synapse analysis identified 13 critical links with the greatest effect on global efficiency, with all nodes being interneurons or motor neurons, and all connections containing at least one interneuron and over ½ at least 1 motor neuron: 6 I → I, 4 I → M, 3 M → I (see red lines in Fig 3E). The interneuron-to-interneuron links are expected to be critical information processing structures in the network, and the others show the significance of motor control. More specifically, two critical pathways were identified, which were again AVA- and PVP-based (Fig 5A and 5B). Thus, the *V*_*E*_ results show that components of both the AVA- and PVP-based pathways are important control segments that have the greatest influence on global network efficiency.

Combining the results for all critical neurons, synapses and vulnerabilities, they appear to converge on three main pathways. These pathways are most clearly defined by the *V*_*B*_ results (Fig 4A), which identify the synapses with the largest effect on network betweenness centrality, the *V*_*E*_ results then highlight the components with the largest effect on global efficiency, and the *V*_*C*_ results show where the most critical clustering occurs. First, there is an AVA-based pathway, beginning with RIB (I) → AVE (I), and ending with either motor neurons or PVC. This pathway strongly matches the backward locomotion control circuit, attesting to the significance of backward locomotion in the *C*. *elegans* nervous system. Second, there is a PVP-based pathway. This pathway is composed of sensory (AQR), interneurons (PVP, DVC, AVH), and a motor neuron (VD01). The sensory to motor structure reveals a complete behavioral circuit, and implicates social feeding behavior. The third critical pathway segment is RMD (M) → OLL (S) that again identifies avoidance control as important—in this case, aversive stimulus avoidance with respect to nose/head. The motor to sensory directional link also highlights the importance of the loop structure in the *C*. *elegans* circuitry [7].

### Critical synapses are also network control structures

To better understand the nature of the critical synapses, we examined the two main local properties of edges: strength (*Str*) (i.e., the weight of the given synapse) and edge betweenness centrality (*EBC*, a betweenness measure for edges; see S1 Text). We first compared the critical and noncritical synapses on *Str* and *EBC* using the Mann-Whitney U test (Fig 4C), and the critical synapses indeed showed significantly higher *Str* and *EBC* than the noncritical synapses. Thus, like the critical neurons, the critical synapses have stronger connections and were those with the most shortest paths running through them—i.e., with the highest volume traffic. As stated previously, constituents with such high volume traffic can be considered as *control structures* by virtue of their influence over the multiple pathways passing through them.

To clarify how *Str* and *EBC* related to each vulnerability measure *V*_*C*,*E*,*B*_, we next examined the correlations (without absolute value for *V*_*C*,*E*,*B*_), and all were statistically significant (Table F in S1 Text). The S1 Text contains a detailed discussion of the results. In sum, like neurons, the synapses most critical for betweenness centrality generally have the highest control themselves, and also tend to link control nodes together. For global efficiency, also like neurons, they generally are those with the highest control in the network, regarding the number of pathways sharing them. There were, however, five exceptions for global efficiency and betweenness centrality (detailed in the S1 Text), and they suggest that, like betweenness centrality, global efficiency can also be critically affected by context. Finally, for clustering (*V*_*C*_), critical synapses appeared to participate in shared projections to neighbors, producing clusters.

## Discussion

Function derives from form, and with unrelenting natural selection, nervous systems must achieve complexity as efficiently as possible. Network analysis provides a normative approach to analyze the solution, and vulnerability analysis helps provide a more experimental and less assumption-laden analysis of individual component contributions to information processing. In this study, we tested the effects of attacks on every neuron and synaptic connection in the *C*. *elegans* connectome to identify and characterize the most critical constituents of the network. To our knowledge, this is the first study to analyze network robustness for individual node and edge attacks on an entire nervous system. We identified 12 neurons and 29 synapses critical for clustering, information integration and propagation.

Although one might have expected the clustering, efficiency, and betweenness values for individual neurons and synapses to be the most important factors determining these same properties at the network level, control structures—i.e., those that influence multiple others—prove the most important. From an evolutionary-development (*evo*-*devo)* perspective, the additions of higher-order control structures can produce the largest effects on information processing, providing the most ‘bang for the buck’. Thus, to most affect clustering, new neurons should project paired synapses to neighbors. To most affect global efficiency, new neurons or synapses should be placed in central positions that shorten the largest number of prior existing pathways. These more centralized neurons not only contribute to overall efficiency and information integration, they also become traffic centers, and thereby have greater influence (and thus control) in the connectome. Finally, to most affect network traffic flow (i.e., global betweenness), new neurons or synapses should provide new pathways that decrease a larger number of path lengths and should also normally link to other control structures, leading to the formation of chains of control units (i.e., node_1_ → node_2_ → node_3_).

Not only were there critical control structures at the cellular level (neurons and synapses), the synapses organized into larger control structures at a higher circuit level—into full or partial circuits. Thus, the vulnerability analysis seemed to uncover fractal-like control in the network [48]. Yet these network control systems must translate to circuits that accomplish the organism’s biological goals, and there was in fact a close correspondence between the identified critical pathways and biological function. The AVA-based pathway is a large component of the backward locomotor circuitry. Identifying this circuitry as a control pathway with respect to its relative influence in the network suggests that there is high volume use of this circuitry. This in turn suggests that backward locomotion likely has multiple trigger stimuli and is utilized by multiple biological functions that require maneuverability. Indeed, given the apparent need for the worm to reverse prior to changing directions (e.g., for omega turns), the finding may highlight the significance of backward movement for navigation more generally. At the same time, to the extent that backward movement reflects the avoidance of particular trigger stimuli, it might also point to the importance of avoidance behavior.

For the PVP-based pathway, given the sensory to motor structure, as well as the AQR neuron being implicated in social feeding and the VD01 neuron in locomotor control, our analysis appears to implicate a social-feeding circuit as a second major critical pathway in the connectome. Moreover, the pathway result implicates PVP, DVC, and AVH involvement in social feeding—neurons whose complete functional roles in information processing in the adult network remain unclear. The third pathway segment has also been implicated in avoidance behavior. Taken together, our results suggest that the network topology is particularly organized around backward movement, avoidance behavior and social feeding, suggesting a primary significance of these functions to the organism. Indeed, backward movement is likely required for maneuverability in multiple behavioral functions. Thus, the *C*. *elegans* appears to have evolved under strong selection pressures for maneuverability, to avoid undesirable and harmful circumstances and to negotiate social interactions. The significance of social behavior would highlight the fundamental importance of behavioral strategies in animals to overcome inevitable competition for resources.

With respect to architectural design, the AVA-based pathway has three general components: a second-layer interneuron (RIB), command neurons (AVE, AVA, PVC), and motor neurons. Thus, it is a subcircuit for information integration, sensorimotor integration, motor control, and motor performance. Moreover, beginning with a second layer interneuron (RIB), it suggests that multiple prior circuit segments may use this critical pathway to produce backward locomotion: for example, for multiple avoidance stimuli to tap into this (backward locomotion) circuit segment. This finding is comparable to Gray et al.’s suggestion of a “common substrate” foraging subcircuit [14]. Such subcircuit structures lend support for a serial-based architectural design structure (i.e., input → central processing → output).

At the same time, our second PVP-based pathway contained a sensory to motor structure that suggests a more complete behavioral circuit, and itself provides evidence for separable behavioral circuits, and thus a parallel design structure [7]. Taken together, our findings point to a mixed or hybrid architectural design. Modularity studies of the *C*. *elegans* have appeared to find evidence for this mixture—with modules aligning both with known biological function to some degree (supporting parallelism), as well as with the input-output serial design structure to some degree (e.g., sensory and first-layer interneurons dominating one module, second-layer interneurons another, and command interneurons and motor neurons another) [8, 28]. Our study highlights and extends these findings by identifying specific neurons, synapses and pathways using a vulnerability approach that implicates specific circuit structures as having critical roles in the connectome. Our findings may also extend those by others who have found significant neural activity across a large extent of the nervous system during activities such as locomotion [26]. Even when such global processing occurs, there is likely a hierarchy of importance or influence among the network components, which can be characterized.

There are multiple avenues for further investigations. For example, future studies should explore other taxa to help determine the evolutionary trajectory of nervous systems by identifying shared and diverging properties with *C*. *elegans*. They could also induce multiple perturbations on the network, such as multiple simultaneous attacks, to help clarify how network components interact to affect network processing properties [31, 49]. In addition, because our findings derive from theoretical analyses, they require empirical verification, especially to test the hypotheses generated from our study: most notably, the functional roles of PVP (sensorimotor integration), DVC (information integration, motor control), and AVH (information integration, motor control) in social feeding behavior (as well as the other possible roles of DVC and AVH derived in the S1 Text).

The nematode *C*. *elegans* is a valuable model system to identify potentially fundamental design elements of nervous systems. Further specification of these principles in the *C*. *elegans* and other nervous systems, including that of humans, will lead to the ultimate goals of a true understanding of nervous system design, and the subsequent medical and technological advances that follow.

## Methods

### *C*. *elegans* connectome

We analyzed the published neuronal wiring data of the nematode *C*. *elegans* hermaphrodite that has information on 279 neurons (pharyngeal and unconnected neurons excluded) with 6,393 chemical synapses and 890 electrical junctions [5, 15, 29]. We examined the directed and weighted full network, generated by combining the gap junction and chemical synapse networks. The weight of a connection between two neurons was defined as the number of gap junctions and chemical synapse contacts between them. Thus, the total number of directed connections, which we call “synapses”, between all neurons were represented as 2,990 synaptic connections with weights.

We have also followed the typical naming conventions used for the connectome. Many neurons have symmetric left, right pairs, which are denoted as “L” and “R”. Thus, for example, the so-called AVA neurons have both AVAL and AVAR counterparts, sometimes written as AVAL/R. Motor neurons are organized into classes and numbered, such that “DA01”, for example, is the first motor neuron of class “DAn”, where *n* represents the number. We denote a synapse with “→”, such as AVAL (I) → AVAR (I), and we put neuron type, sensory (S), interneuron (I), or motor (M), in parenthesis when useful. Finally, to save space we sometimes write *V*_*C*_, *V*_*E*_, and *V*_*B*_ as *V*_*C*,*E*,*B*_.

### Individual attack strategy

We defined an attack on a neuron in the *C*. *elegans* connectome as a deletion of all connections of a target neuron (also called *node*), but the target neuron was still in the adjacency matrix with zero degree. Furthermore, we also defined an attack on a synapse as a deletion of the target connection (also called *edge*). Therefore, 279 neuronal attacked networks and 2,990 synaptic attacked networks were constructed (detailed in the S1 Text Methods). All attacked networks had a corresponding 279 by 279 adjacency matrix that contained the direction and weight edge information, representing the synaptic connections between neurons.

### Isolated nodes, leaves, subnetworks, and reachability

We also examined the connectome’s topological structure with respect to robustness and information propagation potential. These analyses included a characterization of (a) leaf nodes (i.e., ones that have only one connection) in the intact network, (b) isolation from single neuronal attacks based on network fraction (i.e., the extent individual neurons or subnetworks are isolated), and (c) reachability (whether each neuron pair in the connectome has a connected path between them) both in the intact connectome and after neuronal deletions (detailed in the S1 Text).

### Vulnerability

We quantified *vulnerability*, *V*, to determine the effect of the loss of a neuron or synapse on global information processing, as measured by three key graph-theoretic structural properties [50]: the clustering coefficient (*V*_*C*_), global efficiency (*V*_*E*_), and the average betweenness centrality of the network (*V*_*B*_). The vulnerability of a network to a particular loss might also be considered the degree of lethality or redundancy, or the functionality enabled by a component’s addition [35, 36]. *Vulnerability* with respect to each network property was defined as the relative change in value after elimination of the neuron or synapse, namely

$$V_{x}(i)=\frac{\left|X-X(i)\right|}{X},$$ (1)

where *X* is the value of the network property *x* in the original intact network, and *X*(*i*) is the value of the property after the attack of neuron *i*. For each synapse *i* → *j*, (*i*, *j*) was used in place of (*i*).

## Supporting Information

S1 Text Supporting information for “Vulnerability-based critical neurons, synapses, and pathways in the *Caenorhabditis elegans* connectome”. (DOCX)

S1 Fig An example of an undirected circular wiring diagram. (A) Adjacency matrix of an example network. (B) Schematic graph drawing of the example network. (C) Undirected circular wiring diagram.
(TIF)

S2 Fig An example of a directed circular wiring diagram. (A) Adjacency matrix of an example network. (B) Schematic graph drawing of the example network focused on the connections of the node 3. (C) Directed circular wiring diagram.
(TIF)

S3 Fig Directed circular wiring diagram. Link colors show the weights of each connection (light grey: 1–5, grey: 6–10, green: 11–15, blue: 16–20, orange: 21–25, pink: 26–30, and red: over 30). The colors of the names of neurons indicate their neuronal types (sensory neuron: red; interneuron: green; and motor neuron: blue). The lengths of the segments of the outer layer (orange) indicate the overall strength (*Str*) of the nodes. The lengths of the red segments (sinks) indicate the total *Str*_*in*_ of the nodes, and the lengths of the blue segments (sources) indicate the total *Str*_*out*_ of the nodes.
(TIF)

S4 Fig Directed circular wiring diagram sorted by somatic location. Link colors show the weights of each connection (light grey: 1–5, grey: 6–10, green: 11–15, blue: 16–20, orange: 21–25, pink: 26–30, and red: over 30). The colors of the names of neurons indicate their neuronal types (sensory neuron: red; interneuron: green; and motor neuron: blue). The lengths of the segments of the outer layer (orange) indicate the strength (*Str*) of the nodes. The lengths of the red segments (sinks) indicate the total *Str*_*in*_ of the nodes, and the lengths of the blue segments (sources) indicate the total *Str*_*out*_ of the nodes.
(TIF)

S5 Fig Mean *D*, *Str*, *AW*, *C*, *E*, and *B* values for the intact network consisting of all 279 neurons. Bar plots indicate the results of each measure by neuronal types (All: entire 279 neurons; S: 88 sensory neurons; I: 82 interneurons; and M: 109 motor neurons). Error bars represent standard error of the mean. *: *P* < 0.05, **: *P* < 0.01, ***: *P* < 0.001. Interneurons had higher values of degree (*D*, i.e., the number of connections), strength (*Str*, *i*.*e*., the number of connections times their weight), nodal efficiency (*E*(*i*), i.e., the average shortest path between the neuron and all others), and nodal betweenness centrality (*B*(*i*), *i*.*e*., a measure of the degree to which shortest paths travel through the unit) than the other neuronal types. Motor neurons had higher average weight (*AW*, *i*.*e*., the average strength of connections) and nodal clustering coefficient (*C*(*i*), i.e., the degree to which its neighbors are connected with each other) values and lower *E* values than the other neuronal types. Sensory neurons had generally lower *Str* values than the other two.
(TIF)

S6 Fig Mean *D*, *Str*, and *AW* values for all 279 neurons considering directions of the synapses for the intact network. Directional information is indicated as input (*in*) and output (*out*). The ratio of the *out* to the *in* value (*ratio*) is also plotted. Bar plots indicate the results of each measure by neuronal types (All: entire 279 neurons; S: 88 sensory neurons; I: 82 interneurons; and M: 109 motor neurons). Error bars represent standard error of the mean. *: *P* < 0.05, **: *P* < 0.01, ***: *P* < 0.001. The results for both degree and strength (i.e., all connection weights summed) are the same, with interneurons having significantly higher *in* and *out* directions (for both *D* and *Str*) than the other types, motor neurons having significantly higher *in* direction than sensory neurons, and sensory neurons having significantly higher *out* direction than motor neurons. Additionally, sensory neurons have a significantly higher *D*_*ratio*_ (ratio of *out* to *in*) than the other types, while interneurons also have a significantly higher *D*_*ratio*_ than motor neurons. At the same time, motor neurons have significantly higher *AW*_*in*_ than the other types, and interneurons have higher *AW*_*in*_ than sensory neurons (with no significant differences in *AW*_*out*_). Sensory neurons also have a significantly higher *AW*_*ratio*_ than the other types.
(TIF)

S7 Fig Adjacency matrix of the reachability results. The color of an element *a*_*ij*_ depicts the possibility of reachability by network types: i.e., gap junction, chemical synapse, and full networks (pink: the full network only, black: impossible reachability, and white: possible reachability for all networks).
(TIF)

S8 Fig Synaptic connection schemes of the critical neurons whose functions are not known. (A) AVHL and (B) DVC. Biological functions were divided into four categories: (1) blue: body movement or locomotion; (2) red: pioneering, growth, and neuronal development; (3) green: chemical reactions or information integration; (4) gray: unknown function. The number in the parentheses is the count of instances for each specific biological function. Synapse types are also denoted as purple for gap junction and orange for chemical synapse.
(TIF)

S1 File Network properties and vulnerability results for the intact network and the attacked networks for neurons. (XLSX)

S2 File Top 50 neurons for vulnerability measures. (XLSX)

S3 File Network properties and vulnerability results for the intact network and the attacked networks for synapses. (XLSX)

S4 File Top 240 synapses for vulnerability measures. (XLSX)
