# Supplementary material for: Vulnerability-Based Critical Neurons, Synapses, and Pathways in the Caenorhabditis elegans Connectome
Source: PLoS Comput Biol. 2016 Aug 19;12(8):e1005084. doi: 10.1371/journal.pcbi.1005084 (PMC4991803; doi:10.1371/journal.pcbi.1005084)
Supplement: S1 Text — (DOCX) [file pcbi.1005084.s001.docx]

**Supporting Information**

**Vulnerability-based critical neurons, synapses, and pathways in the *Caenorhabditis elegans* connectome**

Seongkyun Kim, Hyoungkyu Kim, Jerald D. Kralik*, and Jaeseung Jeong*

**Methods**

**Single edge attack strategy**

The intact full network was constructed by combining the gap junction and chemical synapse networks. Since the gap junction network is considered an undirected network due to bi-directional ion transfer, we changed the undirected connections to bi-directed connections between neurons to generate the full network. For example, if a connection between neuron *i* and neuron *j* is composed of a gap junction (*i* - *j*), then the connection can be constructed as two directional links *i* 🡨🡪 *j* in the full network. The weight of a specific directional connection between two neurons was defined as the number of gap junctions and chemical synapse contacts between them.

To simulate an attack on an edge *i* 🡪 *j* in the directed and weighted full network, we considered the edge *i* 🡪 *j* to have zero weight after the attack. However, for gap junctions, the fact that a synaptic attack would block bi-directional ion transfer must be properly accounted for. That is, since an edge *i* 🡪 *j* in the gap junction network was represented as two links *i* 🡪 *j* and *j* 🡪 *i* in the full network, if we changed the weight of the edge *i* 🡪 *j* as zero in the full network, the edge *j* 🡪 *i* of the full network would still have a nonzero weight. Thus, this strategy would not fully capture the real biological phenomenon that an attack on a gap junction would block the bi-directional ion transfer. To solve this problem, our strategy for simulating an edge attack was firstly to make an edge attack in both the gap junction and the chemical synapse networks, then combine these two attacked networks to construct an attacked full network.

**Statistical network properties**

To study basic connection properties of weighted networks, we have to consider not only degree, but also the strengths of the edges [1-3]. The node degree *D(i)* is the number of connections of node *i* to its neighbors, and the node strength *Str(i)* is the sum of weights between node *i* and its neighbors [1, 2]. The edge strength *Str*(*i*,*j*) is the weight of the edge from node *i* to node *j*. Although the strength *Str(i)* indicates the total weights of connections of node *i*, it has no information about the relation of degrees and weights [4]. For example, the strength of a node that has 3 degrees with 2 weights is the same as a node that has 6 connections with 1 weight. Thus, to characterize the relation between degrees and weights of individual nodes, we calculated the average weight *AW(i)*, which is the proportion of the strength to the degree of node *i*.

**Circular wiring diagrams**

To draw the circular wiring diagrams, we applied the Circos program [5], which is optimized to visualize sequence similarity and conservation in genomic data. The Circos program is available at <http://www.circos.ca>.

S1 Fig shows an example of an undirected circular wiring diagram consisting of 4 nodes and 6 undirected weighted links. Since the example matrix is undirected (S1A Fig), the schematic graph representation of the matrix is as shown in S1B Fig. In addition, *circular* wiring diagrams offer an easy visualization of the connections of nodes in the adjacency matrix. S1C Fig is the undirected circular wiring diagram of the example matrix. To construct the circular diagram, the strength *Str* value of a node is represented by the length of a segment of the circle. For example, the *Str* value of node 3 of the example matrix is 8 (S1A Fig), and it is represented by the length of the third segment (S1C Fig). In addition, the thickness of a link indicates the weight of the connection between the target nodes.

S1 Fig. An example of an undirected circular wiring diagram.


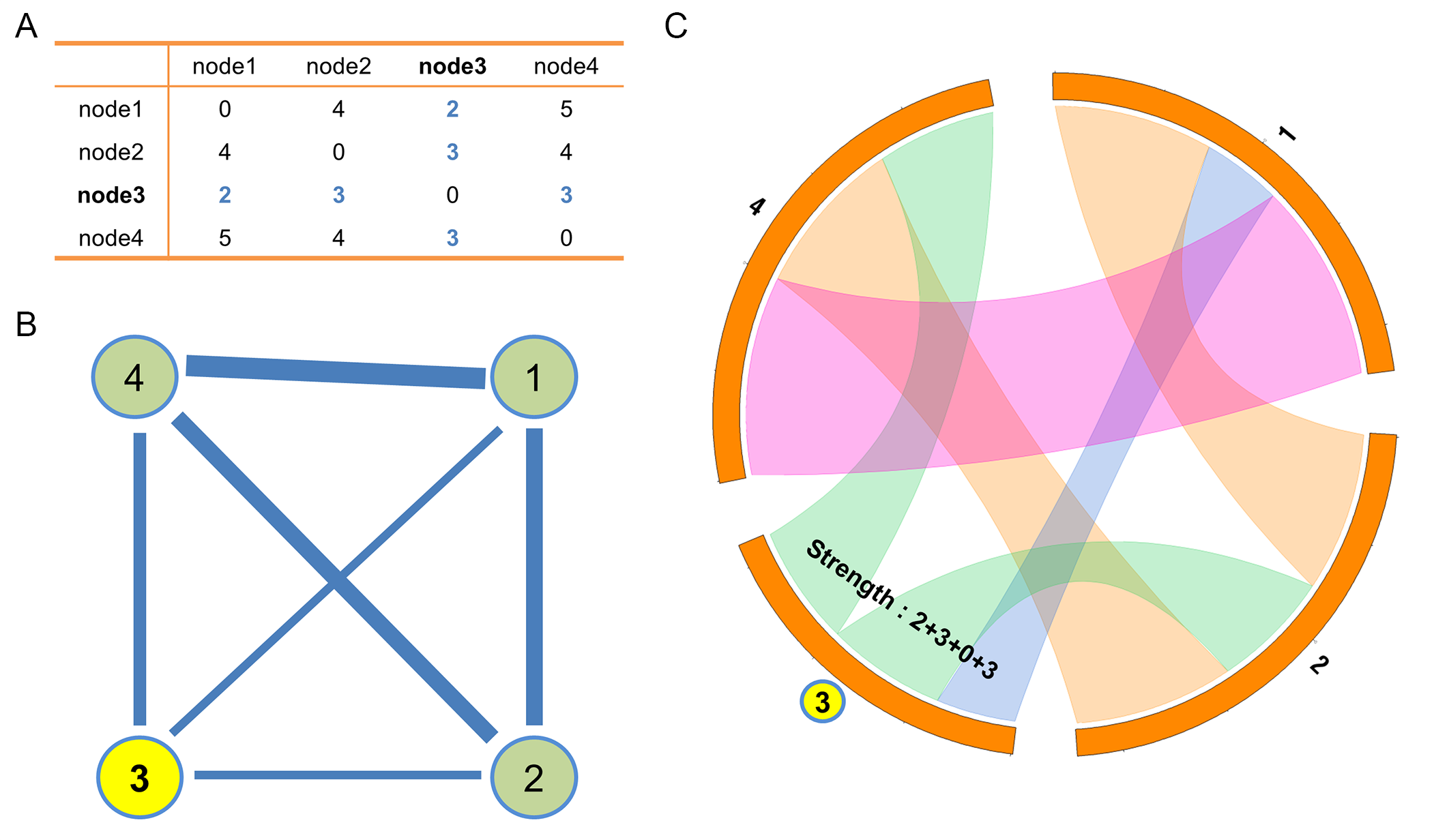


(A) Adjacency matrix of an example network. (B) Schematic graph drawing of the example network. (C) Undirected circular wiring diagram.

However, when creating the directed network diagrams, the directions of links can become problematic. S2B Fig shows how drawing connections from a node (here, node 3) is difficult. To solve this problem in the circular diagram, we constructed one more track (inner layer) to indicate the sources (blue segments) and the sinks (red segments) of the links (S2C Fig). A length of a segment of the inner layer indicates input or output *Str* of a node. A length of a segment of the outer layer indicates the *Str* value of a node.


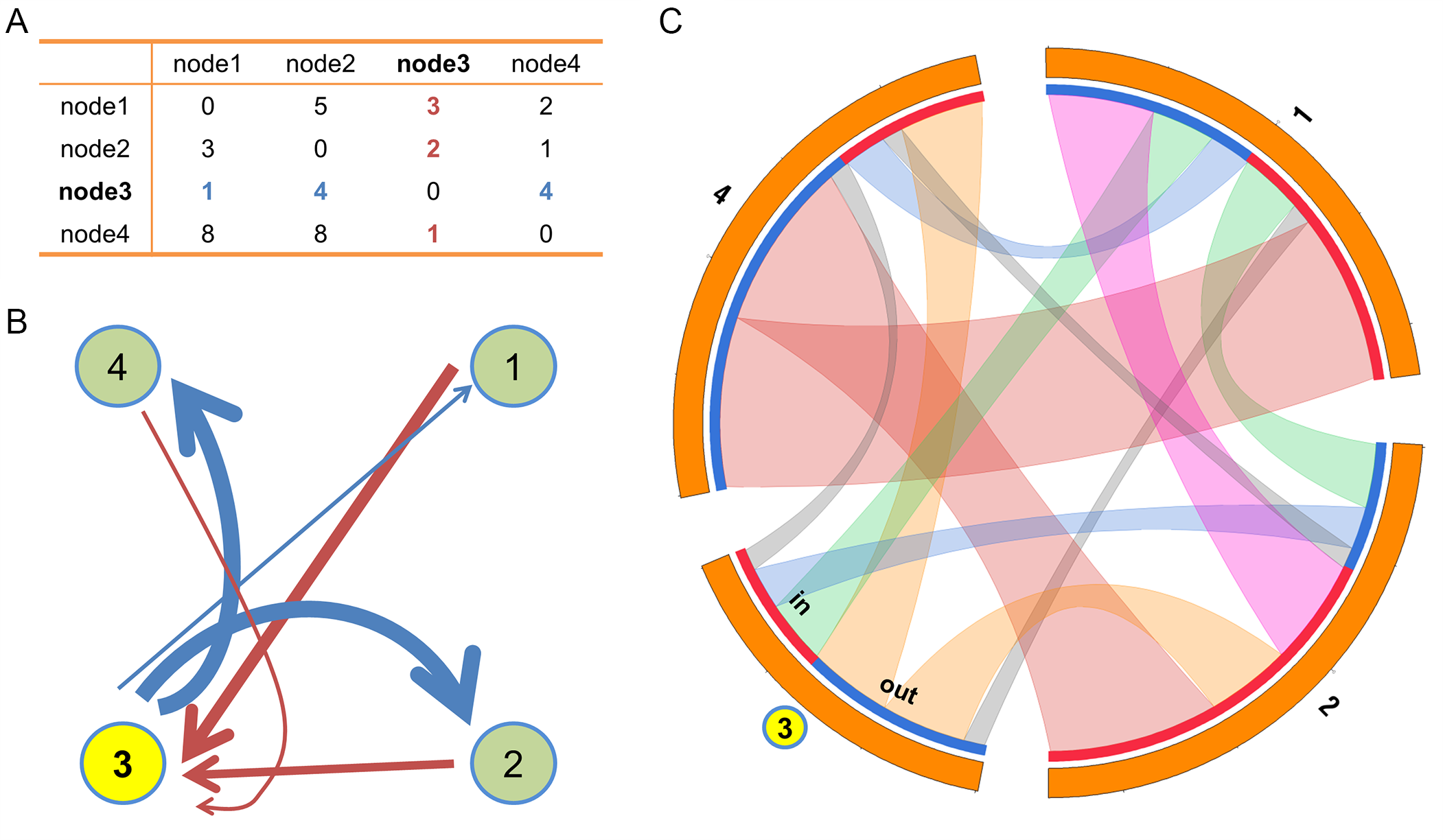


S2 Fig. An example of a directed circular wiring diagram.

(A) Adjacency matrix of an example network. (B) Schematic graph drawing of the example network focused on the connections of the node 3. (C) Directed circular wiring diagram.

**Information processing network properties**

The clustering coefficient (*C*) of a network measures the interconnectivity of neighboring nodes: essentially how many neighbor nodes of a node know each other.

The directed weighted *C* of node *i* is defined as [6],

$\mathrm{C}\text{(}\mathrm{i}\text{)}=\frac{\mathrm{t}^{\mathrm{dw}}\text{(}\mathrm{i}\text{)}}{\text{(}\mathrm{D}_{\mathrm{out}}\text{(}\mathrm{i}\text{)}+\mathrm{D}_{\mathrm{in}}\text{(}\mathrm{i}\text{))(}\mathrm{D}_{\mathrm{out}}\text{(}\mathrm{i}\text{)}+\mathrm{D}_{\mathrm{in}}\text{(}\mathrm{i}\text{)}-1\text{) }-2\sum_{\mathrm{j}\in\mathrm{N}} \mathrm{a}_{\mathrm{ij}}\mathrm{a}_{\mathrm{ji}}},$ (**1)**

where $\mathrm{t}^{\mathrm{dw}}\text{(}\mathrm{i}\text{)}$ is the weighted geometric mean of the directed triangles around node *i,* and $\mathrm{a}_{\mathrm{ij}}$ is a variable that is “1” when there is a link from node *i* to node *j* and “0” when there is no link from node *i* to node *j*.

For a disconnected network, the shortest path length has infinite value when there is no path between two nodes. Since nodal efficiency *E*(*i*), the efficiency of node *i*, is the average of the inverse of the shortest path lengths between its neighbors, isolated nodes have zero efficiency; paths between disconnected nodes were summed to zero for calculating efficiency due to infinite path length. Global efficiency (*E*) is the average of all nodal efficiencies of a network. The *E*(*i*) of node *i* is defined as [7]

$\mathrm{E}\text{(}\mathrm{i}\text{) }=\text{ }\frac{1}{\mathrm{n}-1}\sum_{\begin{matrix} \mathrm{j}\in\mathrm{N} \\ \mathrm{j}\neq\mathrm{i} \end{matrix}} \frac{1}{\mathrm{L}_{\mathrm{i},\mathrm{j}}^{\mathrm{w}}}\text{,}$ **(2)**

where *L^w^_i,j_* is the shortest path length from node *i* to node *j*. Since high synaptic weights means quick paths for flow [4], weights of links are taken as inverse values to calculate *L^w^_i,j_*.

Betweenness centrality (*B*) of node *i* is the number of the shortest paths that pass through node *i* divided by the maximal possible number of connections, defined as [8]

$\mathrm{B}\text{(}\mathrm{i}\text{)}=\frac{1}{\text{(}\mathrm{n}-\text{1)(}\mathrm{n}-\text{2)}}\sum_{\begin{matrix} \mathrm{h},\mathrm{j}\in\mathrm{N} \\ \mathrm{h}\neq\mathrm{j},\mathrm{h}\neq\mathrm{i},\mathrm{j}\neq\mathrm{i} \end{matrix}} \frac{\mathrm{\rho}_{\mathrm{hj}}\text{(}\mathrm{i}\text{)}}{\mathrm{\rho}_{\mathrm{hj}}},$ **(3)**

where *ρ_hj_* is the total number of shortest paths between neuron *h* and *j*, *ρ_hj_*(*i*) is the number of shortest paths between neuron *h* and *j* that pass through neuron *i*. We calculated *C*, *E*, and *B* of a network by averaging the values of the individual network constituents; total number *n* is 279 for all cases – the intact network and the attacked networks.

Edge betweenness centrality (EBC) of edge *i* 🡪 *j* (from neuron *i* to neuron *j*) is defined as the number of shortest paths that pass through synapse *i* 🡪 *j* divided by the maximal possible number of connections, defined as[9]

$\mathrm{EBC}\text{(}\mathrm{i}\text{,j)}=\frac{1}{\text{(}\mathrm{n}-\text{1)(}\mathrm{n}-\text{2)}}\sum_{\begin{matrix} \mathrm{h}\neq\mathrm{j}\in\mathrm{N} \\ \text{(}\mathrm{h}\text{,}\mathrm{j}\text{)}\neq\text{(}\mathrm{i}\text{,}\mathrm{j}\text{)} \end{matrix}} \frac{\mathrm{\rho}_{\mathrm{hj}}\text{(}\mathrm{i}\text{,}\mathrm{j}\text{)}}{\mathrm{\rho}_{\mathrm{hj}}},$ (4)

where *ρ_hj_* is the total number of shortest paths between neuron *h* and *j*, and *ρ_hj_*(*i*,*j*) is the number of shortest paths between neuron *h* and *j* that pass through synapse *i* 🡪 *j*. Again, total number *n* is 279.

**Isolated Nodes, Leafs, Subnetworks, and Reachability**

Because biological function is directly related to the underlying information propagation in the network, we further examined the disturbance of information propagation among neurons induced by single attacks. We defined a *leaf* node as one that has only one connection and an *isolated node* as one that has no connections with neighbors. Since isolated neurons cannot send or receive any information, they should be regarded as losing their function in the network. When a target neuron is connected to a leaf node (that has only one connection), the leaf neuron would be isolated after an attack on the target neuron or after a synaptic attack on this connection.

An important issue in network robustness studies is how a network is split into *subnetworks*. If a network is split into subnetworks due to a single attack, information propagation is possible only in a subnetwork, not the whole network – and thus information propagation is highly affected. This phenomenon thus should be regarded as an isolation of a neuronal group or groups from the whole network. Therefore, the isolation analysis investigated the breaking of the network of the *C. elegans* induced by single nodal or synaptic attack. Furthermore, if an attack induced a loss of all one-directional connections of a neuron or neurons (i.e., all inputs or all outputs), it also could highly disrupt information propagation in the network. Therefore, for the nodal attacks we also examined cases in which all one-directional connections (all inputs or all outputs) were lost.

*Reachability* is the possibility to get information from one node to some other node in a directed network. If neuron *i* can send information to neuron *j* through any route, reachability from neuron *i* to neuron *j* is 1, whereas a zero value indicates a disconnection between two nodes *i* and *j*. First, we calculated the reachability of the *C. elegans* connectome without any attack. To characterize reachability for each synapse type (gap junction or chemical), this analysis was conducted on all three networks: i.e., the gap junction, chemical synapse, and full networks. Second, using only the full network we measured reachability after single neural and synapse attacks, determining which reachability values between neurons changed to zero. Since it is obvious that a targeted neuron in the attack would have no information transmission due to loss of all connections, we did not consider reachability changes between the target neuron and others.

**Results & Discussion**

**General properties of the intact network**

We analyzed the connectome as a combined (i.e., chemical synapses and gap junctions), directed, and weighted network (see Methods and S1 Text Methods). We first examined basic network properties of the intact network.

*Circular wiring diagrams*

We generated circular wiring diagrams for visualization (S1 and S2 Figs), organized by neuron class (sensory, S, interneuron, I, and motor, M) (S3 Fig) and somatic location (S4 Fig). Since the *C. elegans* connectome has bi-directional connections, we added a layer to identify whether the links were sources or sinks (S3 Fig). The neurons in S3 Fig were first sorted with respect to neuronal types (sensory neuron: red; interneuron: green; and motor neuron: blue), then they were sorted by alphabet order within each category. Varshney et al [10] depicted the connection information of gap junction and chemical synapse networks in Table 1 of their paper, and the connection information between neuronal types in the Supporting Tables 2 and 3 of their paper. We believe our study is the first to present a visualization of the connection information of the full network, including connection features across neuronal types. Neurons tend to have strong connections (high *Str*) with the same neuronal types then other types. The AVA neurons showed distinctively large *Str* values, and they are command interneuron as a driver cell for backward locomotion [11]. The AVA neurons tend to have strong connection with all neuronal types, especially to motor neurons. In addition, AVB neurons, functionally rival neurons of the AVA neurons, had high *Str* values that mediate forward locomotion [12]. The AVB neurons tend to have strong connections within specific neuronal types (e.g., interneurons only with other interneurons). Furthermore, in the *C. elegans* connectome, motor neurons have generally high *Str* values of the connections between themselves. PDEL/R 🡪 DVA and OLLL 🡪 AVER have notable *Str* values. The functional role of the PDEL/R 🡪 DVA synapse is modulating locomotion as a response to mechanosensory stimulus from bacteria [13, 14]. The functional roles of the OLLL are related to mechanosensation and pathogen avoidance and the AVER neuron mediates backward locomotion [12, 15]. S4 Fig shows neurons and connections sorted by somatic locations. Neurons in general tended to have strong synaptic connections to physically near neighbors versus far neurons. The neurons in the posterior regions tend to have strong connection with their neighbors than the neurons in the relatively anterior regions. The AVA neurons notably have long-range connections, from head to tail, with high *Str*. These figures indeed highlight the exceptional features of AVA neurons in the *C. elegans* connectome.


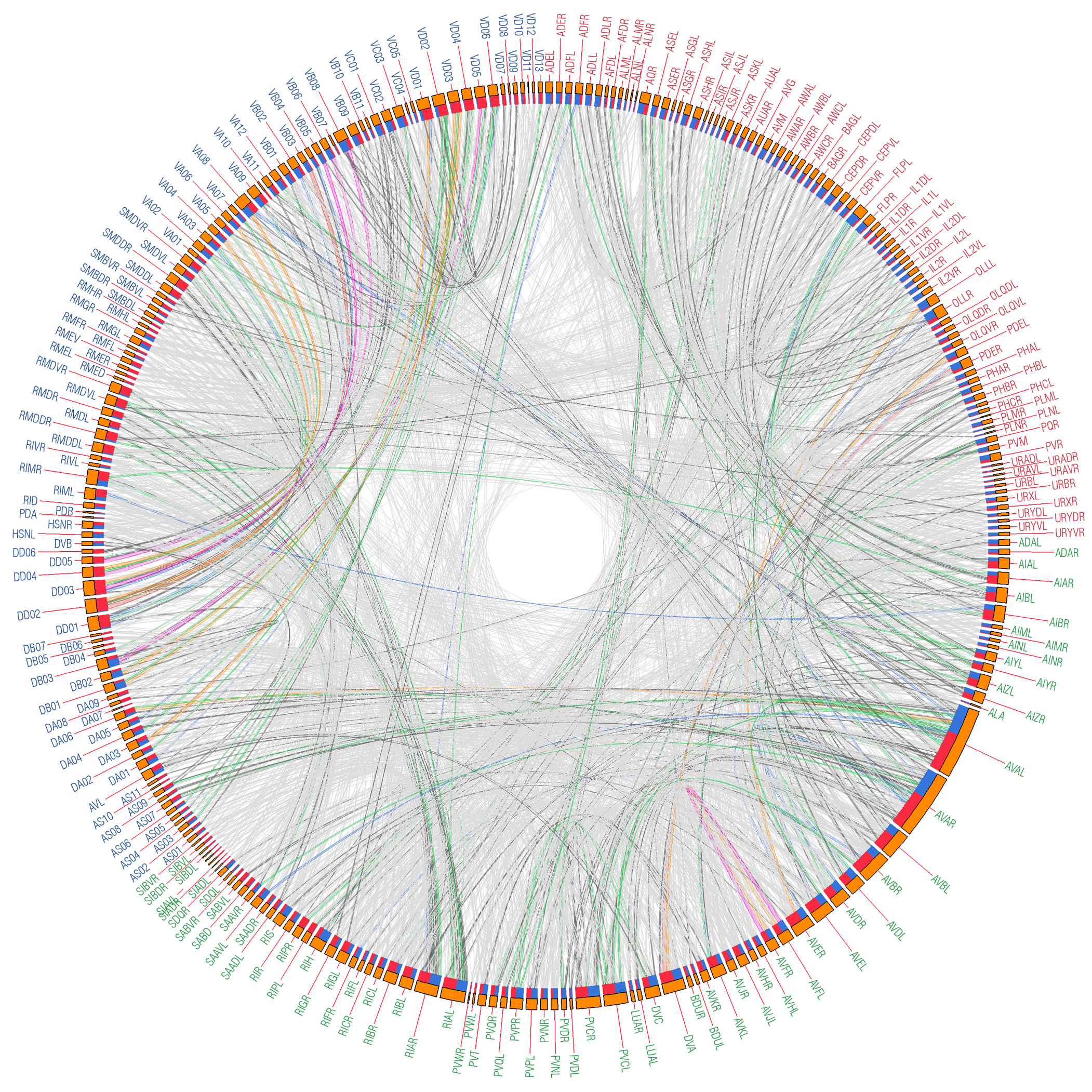


**S3 Fig.** **Directed circular wiring diagram**.

Link colors show the weights of each connection (light grey: 1-5, grey: 6-10, green: 11-15, blue: 16-20, orange: 21-25, pink: 26-30, and red: over 30). The colors of the names of neurons indicate their neuronal types (sensory neuron: red; interneuron: green; and motor neuron: blue). The lengths of the segments of the outer layer (orange) indicate the overall strength (*Str*) of the nodes. The lengths of the red segments (sinks) indicate the total *Str_in_* of the nodes, and the lengths of the blue segments (sources) indicate the total *Str_out_* of the nodes.


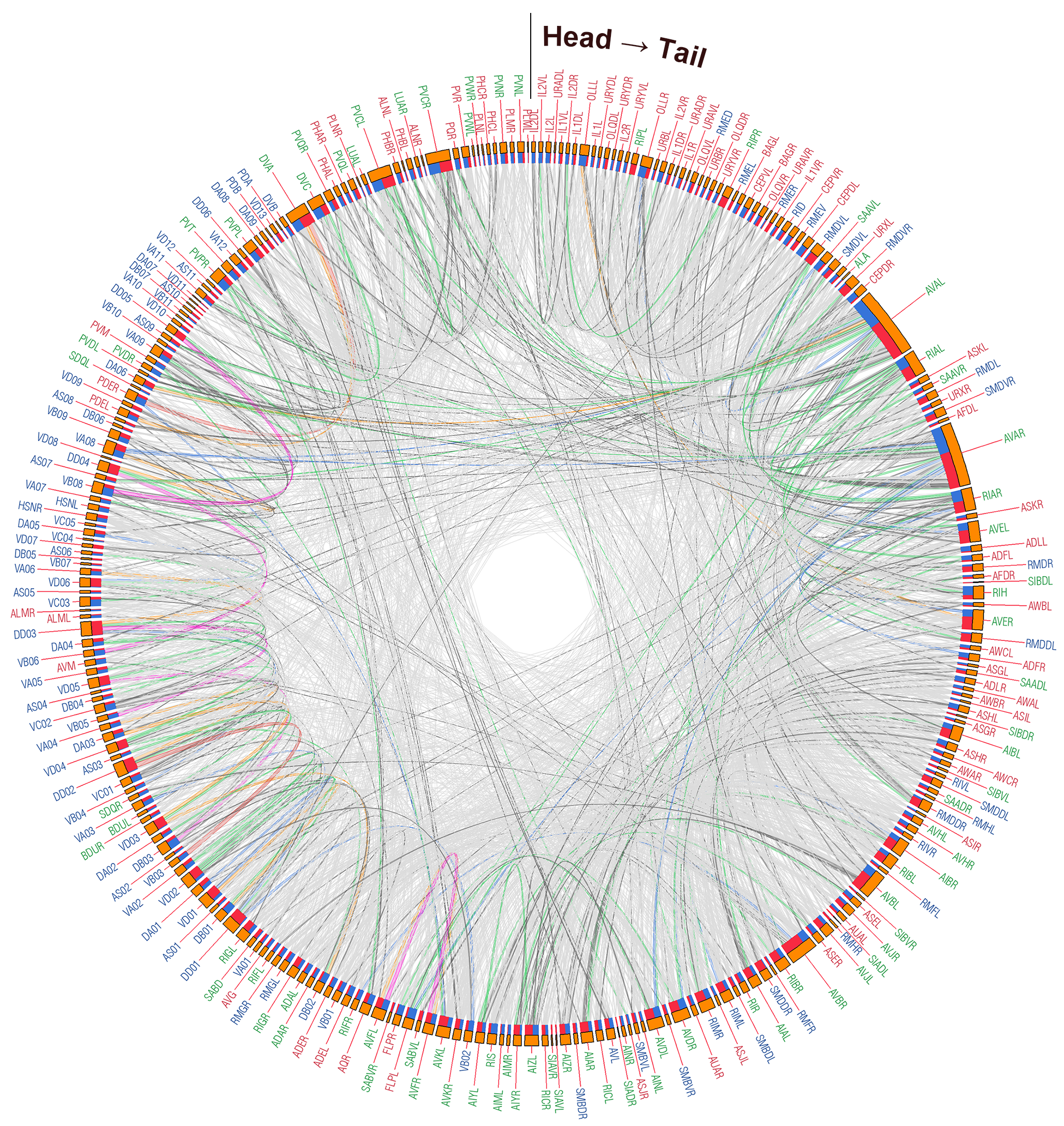


**S4 Fig.** **Directed circular wiring diagram** **sorted by somatic location**.

Link colors show the weights of each connection (light grey: 1-5, grey: 6-10, green: 11-15, blue: 16-20, orange: 21-25, pink: 26-30, and red: over 30). The colors of the names of neurons indicate their neuronal types (sensory neuron: red; interneuron: green; and motor neuron: blue). The lengths of the segments of the outer layer (orange) indicate the strength (*Str*) of the nodes. The lengths of the red segments (sinks) indicate the total *Str_in_* of the nodes, and the lengths of the blue segments (sources) indicate the total *Str_out_* of the nodes.

Overall, the diagrams show high interconnectivity across the connectome, although more specific patterns can also be seen, such as (a) neurons tend to have strong connections with the same neuronal types then other types (e.g., interneurons with other interneurons), and strong connections to physically near neighbors versus far neurons; and (b) distinctively high interconnectivity and strong connections of the AVA neurons, known to participate in the control of backward locomotion.

*Isolated Nodes, Leaves, and Subnetworks*

We calculated the statistical and information processing network properties of all single neurons and synapses with and without an attack (S1 and S3 Files). We conducted a statistic comparison of network properties among neuronal types (sensory versus interneurons versus motor) using one-way analysis of variance (ANOVA). We used Levene's test to estimate the equality of variances among groups. When the result of Levene’s test was positive, Dunnett’s T3 method was performed for *post hoc* comparison within groups. For equal variance (negative result of Levene’s test), we used Tukey’s HSD method.

First, we compared the three general classes of neurons (S, I, and M) on the different network properties (S5 Fig). The results indicate that the interneurons are generally more connected, more strongly and broadly connected, and are important traffic centers compared to the sensory and motor neurons, and thus particularly important for information transmission and integration [10, 16]. Moreover, the results show that the motor neurons have a well-clustered topology with generally strong connections to execute their functions effectively, with fewer more global interconnections than the other types. Finally, the results suggest that the sensory neurons have relatively fewer strong connections, and thus increased specialization.

**
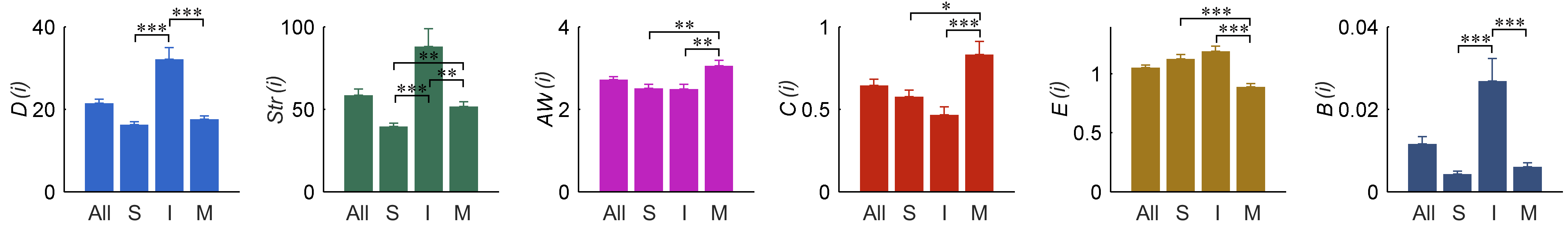
**

**S5 Fig.** **Mean *D*, *Str*, *AW*, *C*, *E*, and *B* values for the intact network consisting of all 279 neurons**.

Bar plots indicate the results of each measure by neuronal types (All: entire 279 neurons; S: 88 sensory neurons; I: 82 interneurons; and M: 109 motor neurons). Error bars represent standard error of the mean. *: *P* < 0.05, **: *P* < 0.01, ***: *P* < 0.001. Interneurons had higher values of degree (*D,* i.e., the number of connections*)*, strength (*Str, i.e*., the number of connections times their weight*)*, nodal efficiency (*E*(*i*), i.e., the average shortest path between the neuron and all others), and nodal betweenness centrality (*B*(*i*)*, i.e.,* a measure of the degree to which shortest paths travel through the unit*)* than the other neuronal types. Motor neurons had higher average weight (*AW, i.e.,* the average strength of connections*)* and nodal clustering coefficient (*C*(*i*), i.e., the degree to which its neighbors are connected with each other) values and lower *E* values than the other neuronal types. Sensory neurons had generally lower *Str* values than the other two.

To examine directional network properties, we tested the *in* and *out* directions separately (S6 Fig). As would be expected, sensory neurons are wired to send more information than receive, interneurons, positioned more centrally in the circuitry, are wired to both receive and send significant amounts of information, and motor neurons to receive more information. In addition, the quality of the inputs increases across the circuit from sensory to interneuron to motor neurons, and highlights the significance of quality over quantity of connections for motor control.

**
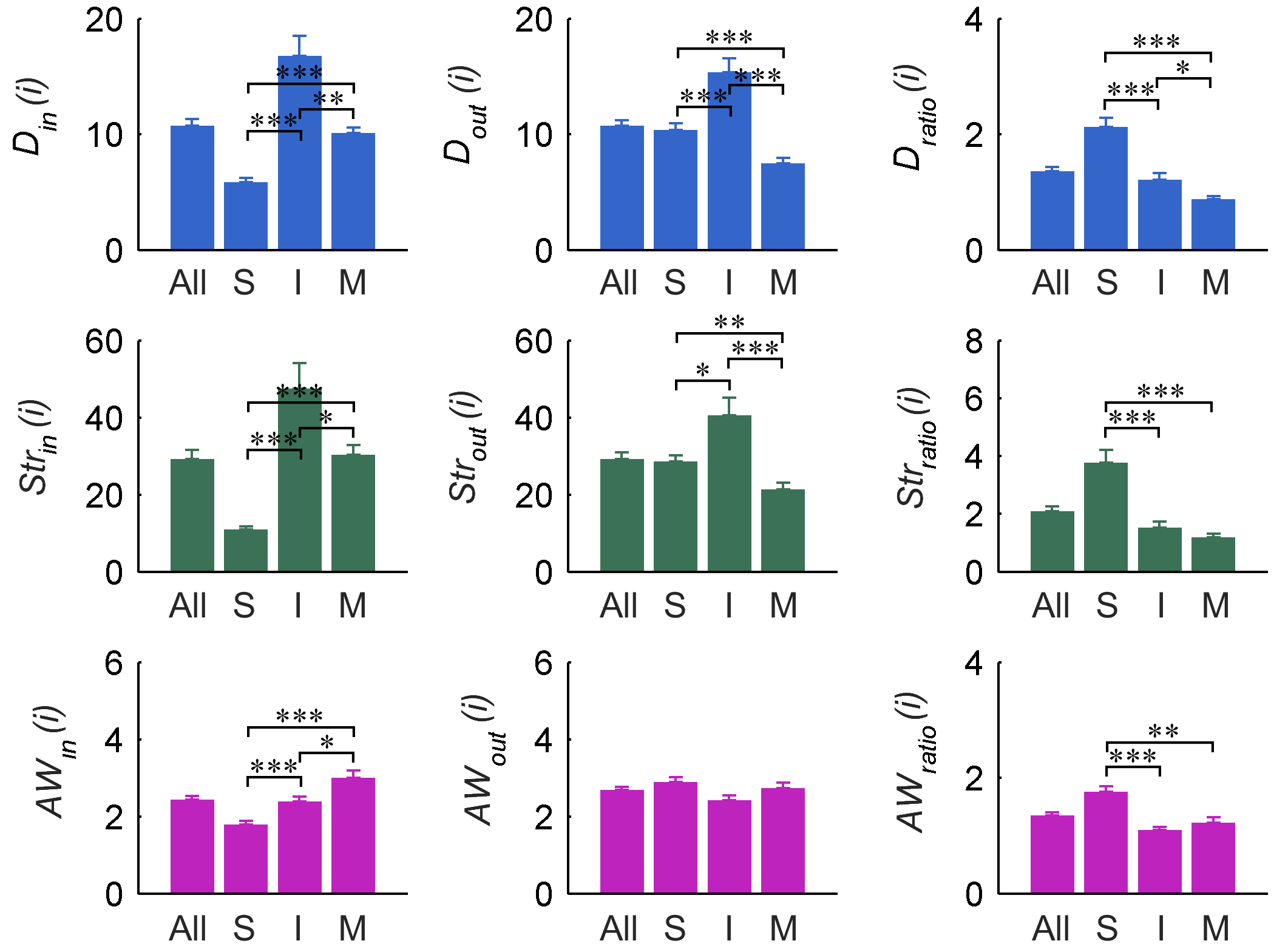
**

**S6 Fig.** **Mean *D*, *Str*, and *AW* values for all 279 neurons considering directions of the synapses for the intact network**.

Directional information is indicated as input (*in*) and output (*out*). The ratio of the *out* to the *in* value (*ratio*) is also plotted. Bar plots indicate the results of each measure by neuronal types (All: entire 279 neurons; S: 88 sensory neurons; I: 82 interneurons; and M: 109 motor neurons). Error bars represent standard error of the mean. *: *P* < 0.05, **: *P* < 0.01, ***: *P* < 0.001. The results for both degree and strength (i.e., all connection weights summed) are the same, with interneurons having significantly higher *in* and *out* directions (for both *D* and *Str*) than the other types, motor neurons having significantly higher *in* direction than sensory neurons, and sensory neurons having significantly higher *out* direction than motor neurons. Additionally, sensory neurons have a significantly higher *D_ratio_* (ratio of *out* to *in*) than the other types, while interneurons also have a significantly higher *D_ratio_* than motor neurons. At the same time, motor neurons have significantly higher *AW_in_* than the other types, and interneurons have higher *AW_in_* than sensory neurons (with no significant differences in *AW_out_*). Sensory neurons also have a significantly higher *AW_ratio_* than the other types.

*Isolated Nodes, Leaves, Subnetworks, and Reachability*

The robustness and information propagation analyses – including characterizing (a) leaf nodes (i.e., ones that have only one connection) in the intact network, (b) isolation from single neuronal attacks (i.e., isolating individual neurons or subnetworks), and (c) reachability (whether each neuron pair in the connectome has a connected path between them) – generally showed that the *C. elegans* connectome is highly robust, both in intact form and in response to single nodal attacks (see Methods in S1 Text; Tables A, B, and C; S7 Fig). For example, there was no network fraction – no subnetwork – induced by a neuronal attack. These results suggest that there is potentially broad information propagation across the network. The specific analyses follow.

***Isolated Nodes, Leaves, and Subnetworks***

Prior to any attacks, the connectome has no isolated neurons. However, IL2DL/R (S), PVDR (S), and PLNR (S) have no input connections with any neighbor, and DD06 (M) is the only one that only has input connections. Although this finding is not unexpected, given that sensory neurons generally obtain information from external stimuli and send it to other neurons, and motor neurons generally receive control signals from other neurons, it nonetheless shows that these particular neurons do not participate in feedback loops.

If a leaf neuron lost its connection because of an attack on its only neighbor neuron, the leaf neuron must become isolated in the network. However, no isolated neurons were produced by the neuron (or synapse) deletions. Nonetheless, there were cases in which node deletions led to neighboring neurons losing all connections of a particular direction (i.e., inputs or outputs), which are listed in Table A. For example, IL2VL has only one input connection from OLQVL. Therefore, the removal of OLQVL produced the loss of all input connections of IL2VL. These results reveal a highly specialized relationship between the specific neuron pairs listed with respect to information propagation in the connectome. Overall, however, since the nodal deletions led to no neural isolations, with only six cases eliminating directional connectivity of only one or two neurons each (listed in the table), it suggests that the *C. elegans* connectome is generally robust with apparently minimal information propagation loss in response to single neuronal or synaptic attacks. Moreover, there was no network fraction – no subnetwork – induced by a neuronal or synaptic attack, again attesting to the general robustness of the network.

**Table A.** The lists of neuron pairs in which a deletion (the first of the pair) causes the second in the pair to lose all inputs or outputs. For example, the removal of OLQVL deleted all input connections of IL2VL. The letters in the parentheses are the abbreviation of neuronal types: S = Sensory neuron; I = Interneuron; M = Motor neuron.

| **No inputs** |
| --- |
| OLQVL (S): IL2VL (S)  SMBVL (M): PLNL (S) |
| **No outputs** |
| RMEV (M): RMER (M)  AVAL (I): DA07 (M)  RIBL (I): SIADL (I),  SIAVL (I)  RIBR (I): SIADR (I),  SIAVR (I) |

***Reachability***

To characterize general connectivity of the connectome via gap junctions, chemical synapses and all synapses together, we analyzed reachability — i.e., the possibility to get from one node to another – in the gap junction, chemical synapse, and full *C. elegans* network. S7 Fig illustrates the reachability results for the intact *C. elegans* connectome. In this adjacency matrix, *a_ij_*, an element in the *i*th row and *j*th column, indicates whether there is a pathway from neuron *i* to neuron *j*. The color of an element *a_ij_* depicts the possibility of reachability from neuron *i* to neuron *j* considering the three network types: i.e., gap junction, chemical synapse, or full network. The pink color indicates that only the full network has possible reachability. The black color indicates that there is no way to propagate information from neuron *i* to neuron *j* in any of the three networks. The white color represented the opposite case of the black color (reachability for all networks). In general, we found multiple instances of unreachability and thus impossible information propagation between two neurons if the connectome only used one particular synapse type (see pink dots). This result indicates that both gap junction and chemical synapses require collaborate with each other to promote information propagation in the *C. elegans* connectome. In addition, this result also supports the validity of the focus on the full network of the *C. elegans* connectome, as we do in the current study.


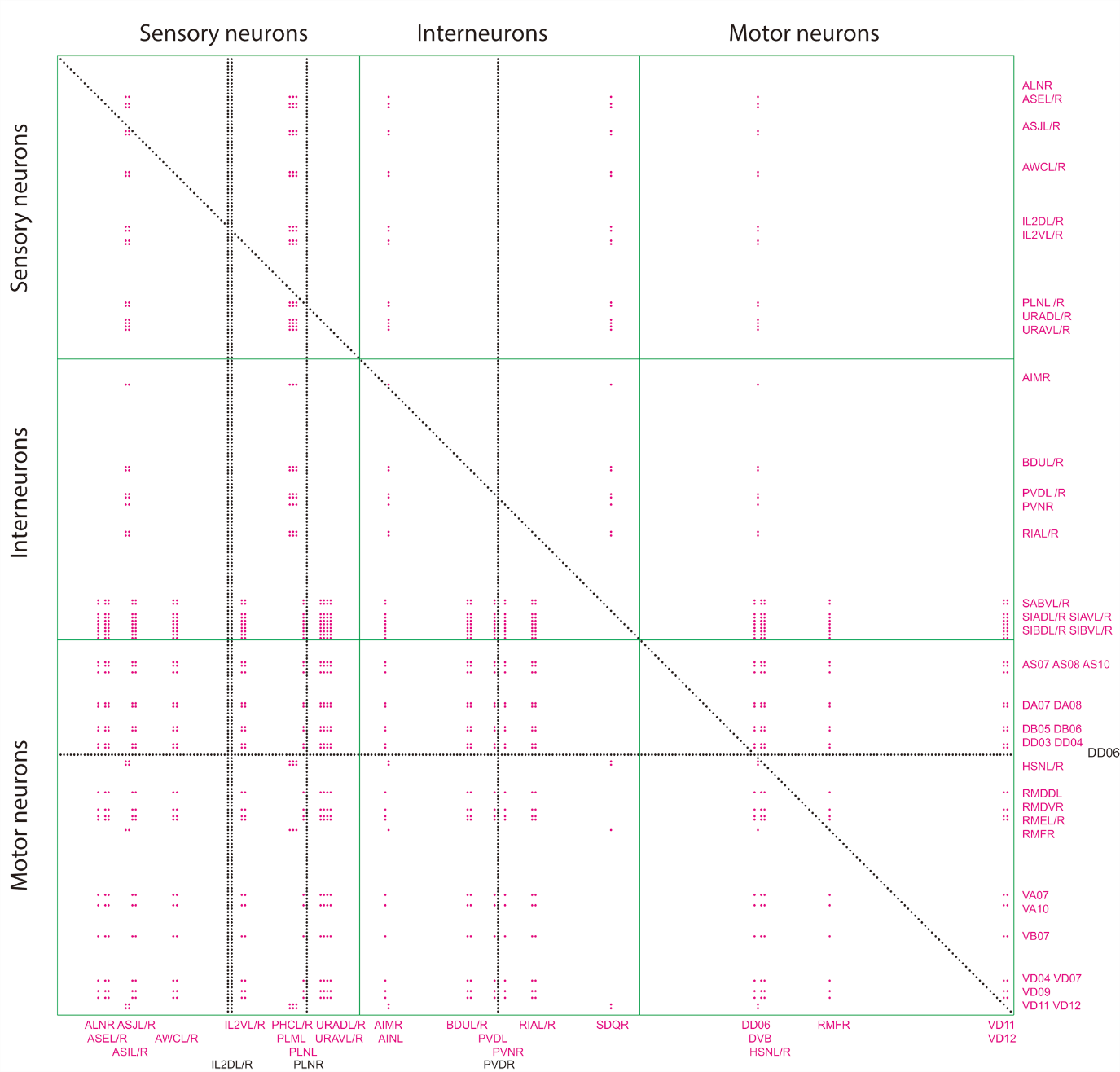


**S7 Fig. Adjacency matrix of the reachability results.**

The color of an element *a_ij_* depicts the possibility of reachability by network types: i.e., gap junction, chemical synapse, and full networks (pink: the full network only, black: impossible reachability, and white: possible reachability for all networks).

We next examined reachability changes after single neuronal and synaptic attacks in the full network. Tables B and C list (a) the neurons (Table B) and synapses (Table C) whose deletions changed the reachability of the network by producing unreachability between certain neuron pairs, (b) the number of disconnected neuron pairs, and (c) a summary of the unreachable neuron pairs. The attacks induced loss of one particular directional connection (no input or no output) of a neuron or neurons. This loss of inputs or outputs by one or two neurons led to disconnections of these neurons with hundreds of others. We summarized these effects as, for example, X 🡪 IL2VL (S), in which IL2VL lost all its input connections due to the attack on OLQVL, leading to a loss in reachability between IL2VL and 276 other neurons represented as X (see Table B). Thus, although the total number of reachability changes were in the hundreds, the specific affected neurons that became disconnected to many others were few (1 or 2). In sum, only 7 neuronal attacks and 15 synaptic attacks changed reachability.

These results suggest two things. On the one hand, because of the relatively small number of neurons or synapses whose deletion affected reachability, and because only one or two neurons were disconnected from the others due to these attacks, it again suggests that information propagation in the *C. elegans* connectome is generally robust against single attacks. On the other hand, even seemingly modest changes in the reachability between nodes induced by single nodal or synaptic attack could nonetheless induce malfunction or late responses because of information processing failures or reroutes. For example, the DA07 motor neuron has two input connections from AVAL/R neurons and has only one output connection to the AVAL neuron. Therefore, if there is an attack on AVAL, DA07 has only two input connections without any output connection (Tables A and B). In addition, if there is an attack on DV07 🡪 AVAL, DA07 has also only input connections from AVAR: since DA07 and AVAL are connected with gap junctions, due to our attack strategy, AVAL 🡪 DA07 is also attacked when DA07 🡪 AVAL is attacked (Table C). Since AVAL and DA07 have functional roles in backward locomotion, this analysis can give helpful information to ablation experimental results involving AVA neurons that the loss of the output connection of DA07 should be a possible cause of abnormal responses in the backward movements [11, 12]. Thus, the reachability results should be considered when interpreting experimental results: deletions not only affect local information transfer, but may lead to wide-reaching functional effects across the connectome. In other words, the reachability results could help identify and lead to a more accurate accounting of the functional circuits.

**Table B.** The list of neurons whose deletions changed the reachability of the *C. elegans* connectome in the full network. The number is the total number of disconnected links between neurons. There were no results for the target neurons, because they obviously could not send or receive any information. The letters in the parentheses are the abbreviation of neuronal types: S: Sensory neuron, I: Interneuron, M: Motor neuron. X indicates arbitrary neuron.

| Target Neurons | # of reachability changes | Summarized changed reachability | |
| --- | --- | --- | --- |
| RIBL (I) | 546 | SIADL (I) 🡪 X, 273 | SIAVL (I) 🡪 X, 273 |
| RIBR (I) | 546 | SIADR (I) 🡪 X, 273 | SIAVR (I) 🡪 X, 273 |
| OLQVL (S) | 276 | X 🡪 IL2VL (S), 276 |  |
| SMBVL (M) | 276 | X 🡪 PLNL (S), 276 |  |
| PVCR (I) | 275 | X 🡪 PVDL (I), 275 |  |
| AVAL (I) | 273 | DA07 (M) 🡪 X, 273 |  |
| RMEV (M) | 273 | RMER (M) 🡪 X, 273 |  |

**Table C.** The lists of synapses whose deletions changed the reachability of the *C. elegans* connectome in the full network. The number is the total number of disconnected links between neurons. The letters in the parentheses are the abbreviation of neuronal types: S: Sensory neuron, I: Interneuron, M: Motor neuron. X indicates arbitrary neuron.

| Target Edges | # of reachability changes | Summarized changed reachability |
| --- | --- | --- |
| OLQVL (S) 🡪 IL2VL (S) | 277 | X 🡪 IL2VL (S), 277 |
| SMBVL (M) 🡪 PLNL (S) | 277 | X 🡪 PLNL (S), 277 |
| PVCR (I) 🡪 PVDL (I) | 276 | X 🡪 PVDL (I), 276 |
| AVAL (I) 🡪 DA07 (M) | 274 | DA07 (M) 🡪 X, 274 |
| DA07 (M) 🡪 AVAL (I) | 274 | DA07 (M) 🡪 X, 274 |
| RIBL (I) 🡪 SIADL (I) | 274 | SIADL (I) 🡪 X, 274 |
| RIBL (I) 🡪 SIAVL (I) | 274 | SIAVL (I) 🡪 X, 274 |
| RIBR (I) 🡪 SIADR (I) | 274 | SIADR (I) 🡪 X, 274 |
| RIBR (I) 🡪 SIAVR (I) | 274 | SIAVR (I) 🡪 X, 274 |
| RMER (M) 🡪 RMEV (M) | 274 | RMER (M) 🡪 X, 274 |
| RMEV (M) 🡪 RMER (M) | 274 | RMER (M) 🡪 X, 274 |
| SIADL (I) 🡪 RIBL (I) | 274 | SIADL (I) 🡪 X, 274 |
| SIADR (I) 🡪 RIBR (I) | 274 | SIADR (I) 🡪 X, 274 |
| SIAVL (I) 🡪 RIBL (I) | 274 | SIAVL (I) 🡪 X, 274 |
| SIAVR (I) 🡪 RIBR (I) | 274 | SIAVR (I) 🡪 X, 274 |

**Unknown biological functions**

Although the *C. elegans* connectome has well-known structure and neuronal functions, a number of functionally unknown neurons remain. For the critical constituents, it is important to consider their biological function. During this project, AVHL and DVC were conspicuous critical neurons whose biological function had been unknown. There has since been new evidence for DVC involvement in locomotion [17], although we also note that due to the integrative nature of the *C. elegans* connectome, it is possible that many neurons (especially interneurons) participate in multiple functions. In the main manuscript, we suggested possible biological functions of AVH and DVC neurons by considering the main *critical* *pathways* uncovered by our individual synapse attack analysis. Here we also consider the possible functions of AVHL and DVC neurons by conducting an additional more local analysis focusing on the biological functions of their neighbors (including all neighbor connections, those with the highest connection strength, and critical connections with respect to vulnerability), as well as reachability. Although this analysis derives a larger set of possible biological functions, it could provide helpful direction for and understanding of future experimental studies (e.g., laser ablation work), as well as help guide future research focusing on multifunctionality of individual components in the network.

First, to produce S8 Fig, we arranged all neighbors of the AVHL and DVC neurons by using the directional information and the synaptic types of the connections. We then listed all known functions of the neighbors. We consider all functions listed in S8 Fig to be possible functions of the AVHL and DVC neurons. For the AVHL interneuron, these include seven instances that suggest involvement in body movement or locomotion (S8A Fig, blue circles); four in pioneering, growth, and neuronal development (S8A Fig, red circles); and six in chemical reactions or information integration (S8A Fig, green circles). For the DVC interneuron, these include nine instances that suggest involvement in body movement or locomotion (8B Fig, blue circles); four again in pioneering, growth, and neuronal development (S8B Fig, red circles); and seven in chemical reactions or information integration (S8B Fig, green circles).

To better triangulate possible function, we next examined several specific characteristics that could help generate a weighted list that reflects the likelihood (or degree) of possible functions of AVHL and DVC. In all cases we only considered the neurons in which evidence for biological function is available. We first considered the number of neighbors with evidence for a particular biological function. For AVHL, the top three biological functions in terms of number of neighbors with evidence for the function were locomotion (three instances), ventral cord pioneering (i.e., first during development for axon guidance) (three instances), and chemorepulsion (two instances) (with all other functions having one instance each). For DVC, the top two functions were locomotion and ventral cord pioneering (three instances each), with several others having two or one instance each (S8 Fig).

We next reasoned that the neighbors with higher *Str* should have a higher probability (or degree) of shared biological function with the AVHL and DVC neurons. AVHL has particularly strong connections with ADFR, AIML, PVPR, PVQR, and SMBVR neurons (S8 Fig). Thus, the *Str* examination for AVHL again provides evidence for all three main categories of biological function, and in particular, locomotion (SMBVR), ventral cord pioneering (PVPR and PVQR), and serotonin related responses including chemotaxis (ADFR and AIML) and swim initiation (AIML) (S8 Fig). For DVC, there are strong connections with AVAL/R, AVL, DVB, VD01, VD10, and PVPR neurons (S8 Fig). Therefore, the *Str* examination for DVC again provides further evidence for all three main categories, and more specifically, locomotion especially related to backward (AVAL/R) and sinusoidal body movement (VD01 and VD10), ventral cord pioneering (PVPR), and defecation (AVL and DVB) (S8 Fig).

Critical synaptic connections should also have important functional role in the neuronal circuits, and thus we next considered the criticality findings from the vulnerability analysis. AVHL has a critical synaptic input from the PVPR neuron (Table 2). Therefore, ventral cord pioneering is again pointed to for the AVHL neuron. For DVC, there are two critical synaptic connections with the VD01 and PVPR neurons (Table 2); thus, sinusoidal body movement (VD01) and ventral cord pioneering (PVPR) are again pointed to.

We also reasoned that a change in reachability could be a possible clue to possible function. Although only in the gap junction network (not in the full network), an attack on the AVHL induced a loss of information propagation of the PHB sensory neurons whose functional role is chemorepulsion [18] (if AVHL was attacked in the gap junction network, there were 490 reachability changes in the gap junction network; PHB - X). This result may suggest that AVHL is involved in chemorepulsion; and thus, the reachability results also appear to point to chemotaxis.

Finally, considering relevant experimental findings in the literature, previous studies of the DVC neuron using laser ablation or mutant studies also corroborate the predictions generated from our analysis. Durbin’s previous study found that PVQR flattens out when the DVC neuron was removed [19]. A well-established functional role of the PVQR is related to pioneering. Therefore, this result further supports the suggestion that the DVC neuron should have a functional role in ventral cord pioneering. Finally, there are also two previous studies that support the conjecture that the DVC neuron should have a functional role in locomotion. Chao and colleagues found that the genetic expression mosaic of *lin-12* induced an abnormal spontaneous reversal rate variant during locomotion [20]. In addition, Ardiel and Rankin suggested that the DVC neuron could have a functional role in backward movement as a stretch receptor [17] (we note that the Wormatlas hompage [12] lists only Ardiel and Rankin’s suggestion [17] for the functional role of the DVC neuron).

Taken together, our biological function analysis focusing on neighbors (all connections, most instances, strongest, and most critical connections), reachability, and literature findings lead to the following hypothesized functions. For AVHL, all functions listed in S8A Fig are possible, with ventral cord pioneering (from number of neighbors, *Str*, and critical connections examination), chemotaxis (from number of neighbors, *Str* and *reachability* examination), and locomotion (from number of neighbors and *Str* examination) being particularly highlighted. For DVC, again all functions listed in S8B Fig are possible, with locomotion, especially backward and sinusoidal movement, and ventral cord pioneering (from number of neighbors, *Str*, critical connections, and previous literature examination), as well as defecation (from *Str* examination) being particularly highlighted. Because our analysis identified potential involvement in multiple biological functions for each neuron (AVHL and DVC), it again suggests that interneurons in the *C. elegans* connectome appear to participate in multiple functions, likely reflecting the highly integrative nature of the network, and providing evidence for an integrated and serial architectural design structure (i.e., input 🡪 central processing 🡪 output). We also note that although ventral cord pioneering is important for axon guidance during neuronal development of the *C. elegans* connectome, if these neurons remain integrated in the connectome in the fully developed adult, they likely participate in functions other than ventral cord pioneering, again attesting to the likelihood of multifunctionality in these neurons. In any case, we hope our analysis approach and results will help provide guidance in experimental design and interpretation of results for future studies identifying biological function of neurons and synapses in the *C. elegans* nervous system (and in particular, for AVHL and DVC).


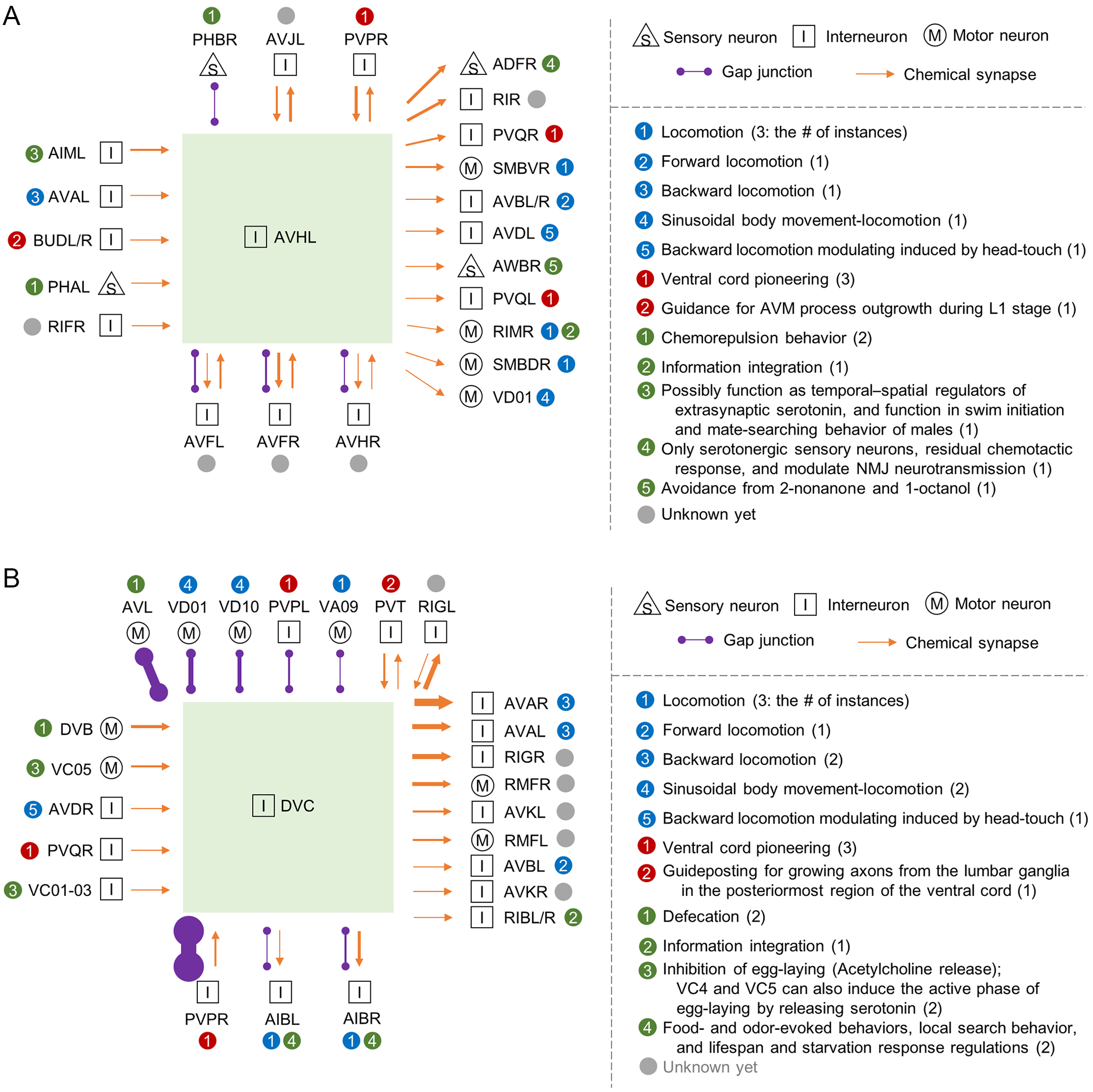


**S8 Fig. Synaptic connection schemes of the critical neurons whose functions are not known.**

(A) AVHL and (B) DVC. Biological functions were divided into four categories: (1) blue: body movement or locomotion; (2) red: pioneering, growth, and neuronal development; (3) green: chemical reactions or information integration; (4) gray: unknown function. The number in the parentheses is the count of instances for each specific biological function. Synapse types are also denoted as purple for gap junction and orange for chemical synapse.

**Attacked network properties**

See main manuscript.

**Table D.** Network properties of the 12 critical neurons.

| Neuron | Criticality | | | | Intact network property | | | | | | Vulnerability | | |
| --- | --- | --- | --- | --- | --- | --- | --- | --- | --- | --- | --- | --- | --- |
|  | *C* | *E* | | *B* | *D*(*i*) | *S*(*i*) | *AW*(*i*) | *C*(*i*) | *E*(*i*) | *B*(*i*) | *V_C_*(*i*) | *V_E_*(*i*) | *V_B_*(*i*) |
| AQR |  |  | | 0 | 29 | 84 | 2.897 | 0.732 | 1.410 | 0.046 | 0.00479 | 0.01297 | 0.01784 |
| AVAL | 0 | 0 | | 0 | 134 | 606 | 4.522 | 0.284 | 2.096 | 0.299 | 0.13576 | 0.06744 | 0.01752 |
| AVAR | 0 | 0 | |  | 137 | 563 | 4.109 | 0.286 | 1.948 | 0.230 | 0.15175 | 0.05423 | 0.00227 |
| AVER |  |  | | 0 | 63 | 176 | 2.794 | 0.306 | 1.664 | 0.094 | 0.00323 | 0.02538 | 0.01938 |
| AVHL |  |  | | 0 | 30 | 57 | 1.900 | 0.393 | 1.128 | 0.036 | 0.00058 | 0.00939 | 0.02117 |
| DA01 |  |  | | 0 | 19 | 70 | 3.684 | 1.214 | 1.417 | 0.050 | 0.00691 | 0.01556 | 0.01936 |
| DVA |  | 0 | |  | 59 | 190 | 3.220 | 0.300 | 1.555 | 0.126 | 0.00030 | 0.02988 | 0.00098 |
| DVC |  |  | | 0 | 37 | 128 | 3.459 | 0.461 | 1.684 | 0.107 | 0.00190 | 0.02306 | 0.02552 |
| PVCL |  | 0 | |  | 64 | 202 | 3.156 | 0.497 | 1.514 | 0.178 | 0.01502 | 0.02962 | 0.00391 |
| PVCR |  | 0 | |  | 69 | 211 | 3.058 | 0.454 | 1.770 | 0.073 | 0.01890 | 0.02708 | 0.00187 |
| PVPR |  |  | | 0 | 35 | 106 | 3.029 | 0.484 | 1.638 | 0.078 | 0.00203 | 0.01595 | 0.02282 |
| VD01 |  |  | | 0 | 27 | 108 | 4.000 | 0.556 | 1.195 | 0.076 | 0.00341 | 0.01686 | 0.01779 |
| For all 279 neurons | | | Mean | | 21.434 | 58.552 | 2.715 | 0.643 | 1.055 | 0.012 | 0.00507 | 0.00869 | 0.00741 |
|  |  |  | SD | | 16.858 | 60.162 | 1.158 | 0.630 | 0.351 | 0.029 | 0.01269 | 0.00609 | 0.00315 |

**Table E.** Rank of network properties of the 12 critical neurons.

| Neuron | Criticality | | | Intact network Property | | | | | | Vulnerability | | |
| --- | --- | --- | --- | --- | --- | --- | --- | --- | --- | --- | --- | --- |
|  | *C* | *E* | *B* | *D*(*i*) | *S*(*i*) | *AW*(*i*) | *C*(*i*) | *E*(*i*) | *B*(*i*) | *V_C_*(*i*) | *V_E_*(*i*) | *V_B_*(*i*) |
| AQR |  |  | 0 | 47 | 47 | 95 | 73 | 41 | 15 | 81 | 30 | 6 |
| AVAL | 0 | 0 | 0 | 2 | 1 | 21 | 225 | 1 | 1 | 2 | 1 | 8 |
| AVAR | 0 | 0 |  | 1 | 2 | 31 | 223 | 2 | 2 | 1 | 2 | 272 |
| AVER |  |  | 0 | 7 | 11 | 109 | 214 | 13 | 6 | 120 | 6 | 4 |
| AVHL |  |  | 0 | 40 | 103 | 212 | 173 | 120 | 22 | 240 | 71 | 3 |
| DA01 |  |  | 0 | 113 | 67 | 52 | 25 | 38 | 13 | 49 | 19 | 5 |
| DVA |  | 0 |  | 10 | 9 | 73 | 217 | 21 | 4 | 262 | 3 | 278 |
| DVC |  |  | 0 | 28 | 15 | 65 | 137 | 12 | 5 | 172 | 8 | 1 |
| PVCL |  | 0 |  | 6 | 7 | 75 | 127 | 25 | 3 | 12 | 4 | 254 |
| PVCR |  | 0 |  | 5 | 5 | 81 | 142 | 8 | 10 | 8 | 5 | 276 |
| PVPR |  |  | 0 | 31 | 29 | 84 | 130 | 17 | 8 | 162 | 17 | 2 |
| VD01 |  |  | 0 | 61 | 28 | 34 | 109 | 99 | 9 | 114 | 14 | 7 |

**Correlations between intact network properties and Vulnerability**

To examine the correlation between the graph-theoretical values of the intact network prior to attacks and vulnerability after attacks, Pearson’s correlation coefficients were calculated (Table F). We first describe the findings for the neurons, and then for the synapses.

*Neuron results*

For *V_C_*, analysis with neurons we found that all network properties correlated significantly with it, with *Str* (which reflects both degree and weight) showing the strongest correlation (0.734), followed by *B*(*i*) (0.698) and *D* (0.510). Thus, neurons with multiple strong connections (*Str*), as well as having the most shortest paths traveling through them (*B*(*i*)) tend to connect to neurons that are neighbors of each other, producing clustering (i.e., interconnections among a given neuron’s neighbors). This effect is best exemplified in the command interneurons (e.g., AVA), in which they tend to coordinate processing among motor neurons that are neighbors. It is interesting to note that, in fact, *C*(*i*) correlated the least strongest with *V_C_* (0.243), providing an example of the difference between local and global network influences. Thus, the neurons critical for network clustering (AVAL/R) do not isolate into clusters themselves, but create clusters with coordinated projections.

For *V_E_*, all nodal network properties were correlated with it except for *C*(*i*), with *B*(*i*) (0.935) the most, followed by *Str* (0.909), *D* (0.781), *E*(*i*) (0.539), and *AW* (0.329). Thus, neurons that provide a shortest path between multiple other neurons (i.e., with high *B*(*i*)) will most strongly affect overall path lengths (*V_E_*) in the network if they are lost—more so than losing the neurons with the shortest average path lengths (*E*(*i*)). Noting that nodal betweenness centrality *B*(*i*) is a form of control by virtue of the component’s influence on multiple pathways sharing it, the critical *V_E_* neurons were therefore those with a larger degree of control [21]. In other words, the neurons most critical for global efficiency are not ones with high nodal efficiency *E*(*i*) themselves *per se*, but rather *control structures* that more strongly influence global efficiency by virtue of their influence over multiple underlying pathways. These critical control structures for *V_E_* also tended to be more centralized in the connectome, and in fact, all were identified as interneurons.

Finally, for *V_B_*, there were three significant correlations: *Str* (-0.374), then *D* (-0.363), then *B* (-0.255). Note that the negative relationships mean that the node’s removal led to higher overall network betweenness; increased *X_B_*(*i*) (higher than *X_B_*) made *V_B_*(*i*) negative (without taking the absolute values). Overall, average network betweenness centrality is influenced by the change in nodal betweenness centrality due to redirecting of traffic through alternative routes (detours), as well as the loss in nodal betweenness centrality of the attacked node and the losses generated in others due to the loss of the attacked connections. When average network betweenness centrality *X_B_*(*i*) increased due to an attack (*V_B_*(*i*) had a negative value), it generally meant that longer detours were necessary for the redirected traffic; and when average network betweenness centrality decreased, it generally meant that betweenness centrality losses outweighed any other increases due to traffic redirection. We observed both positive and negative changes in the dataset prior to taking the absolute values of the vulnerabilities. Overall, however, the neuronal losses tended to lead to longer detours, leading to a negative correlation with *V_B_*. The fact that *Str* and *D* were the most highly correlated to *V_B_* suggests either (a) that the most connected nodes also have the greater likelihood of having multiple connections that are important highway route segments in the connectome; or (b) that the complexity inherent in betweenness centrality, in which the effect of neuronal loss depends on the topological context, may lead to a complex nonlinear relationship not well captured by the linear correlations.

In fact, upon inspection of neuron rankings on nodal betweenness centrality *B*(*i*) of the 8 critical neurons for *V_B_*, 5 were in the top 10 for *B*(*i*), and all 8 were in the top 22 (279 total neurons) (Tables D and E). These critical *V_B_* neuron rankings were much stronger than for *D* and *Str*, with the range for *D* being top 2-113, and for *Str,* top 1-103 (Tables D and E). In comparison, for *V_E_*, which showed the strongest correlation to *B*(*i*), of the 5 critical neurons for *V_E_*, 4 are the top 4 for *B*(*i*), and the fifth was the 10^th^ highest (Tables D and E). Although appearing stronger for *V_E_*, the rankings still suggest a strong relationship between *B*(*i*) and *V_B_* that is likely not captured well by the linear correlation. This complex relationship between *B*(*i*) and *V_B_* warrants further analysis in future studies.

The *V_B_* analysis shows that a neuron’s importance to betweenness centrality is contextual. For neurons critical for *V_B_*, 7 of 8 (except for AVAL) exhibited average network betweenness centrality decrease (*V_B_* prior to taking the absolute value had positive values) when removed. This suggests that the losses in nodal betweenness centrality of the attacked node and other nodes affected by the loss were greater than any potential nodal betweenness centrality increases. The largest losses appeared to particularly occur when the node was connected to other nodes with high nodal betweenness centrality. Thus, the critical nodes for *V_B_* tended to be connected to other control structures.

*Synapse results*

For synapses, to clarify how *Str* and *EBC* related to each vulnerability measure *V_C,E,B_*, we next examined the correlations (without absolute value for *V_C,E,B_*), and all were statistically significant (Table F). For *V_C_*, *Str* (r=0.257) was more influential than *EBC* (0.117). Thus, more strongly connected neurons tended to share neighbors (i.e., cluster). This result appears to reflect the previous finding that the clustering coefficient *C*(*i*) and the average connection weight *AW* were higher for motor neurons, and thus interrelated. In addition, *EBC* also showed some relation to *V_C_*, suggesting that the two nodes of a synapse with high *EBC* also tended to share neighbors. This pattern is again best exemplified in command interneuron (e.g., AVA) connections to motor neuron neighbors. In contrast, for both *V_E_* and *V_B_*, *EBC* (*V_E_*: 0.726; *V_B_*: 0.205) was more influential than *Str* (*V_E_*: 0.468; *V_B_*: 0.119). This result for *V_E_* is similar to that found for the critical neurons, showing again that when a link that provides a lowest path for multiple neurons is lost (high *EBC*), it has a larger overall effect on the network (and since higher *Str* is by definition a shorter path, *V_E_* was affected by *Str* as well). These links were also more centralized, with all being interneuron-interneuron or interneuron-motor links, which would be expected to be critical control structures in the network. A similar effect of edge betweenness centrality on global efficiency was found by Kaiser and Hilgetag [22] for several other kinds of complex networks, including both macaque and cat cortex. We extend the finding here to the *C. elegans* connectome. The *V_B_* results for critical synapses differed from those for the neurons in both the order (betweenness centrality more strongly related than strength) and the direction (positive, and thus, synapse loss decreasing average network betweenness centrality: *X_B_*(*i,j*) – *V_B_*(*i*,*j*) had positive values) of the relationships.

Although there was a significant correlation of *EBC* to *V_B_*, we again considered whether the relationship was actually higher than revealed by the correlation. Examining the synapse rankings for *EBC*, for the 17 critical synapses for *V_B_*, 10 were in the top 13, 12 in the top 20 (the other 5 ranged between top 63-1001) (out of 2990 total synapses) (Tables G and H). To compare to *V_E_*, which showed the highest correlation to *EBC*, for the 13 critical synapses for *V_E_*, 7 are in the top 8, 8 in the top 15 (with the other 5 ranging between top 35-335) (Tables G and H). Since the *V_B_* rankings for *EBC* are comparable to *V_E_*, especially the top 12 of 17 for *V_B_*, it suggests that there is a stronger relationship between *EBC* and *V_B_* that is not well captured by the linear correlation.

Because the *EBC* correlation with *V_B_* was positive, and 15 of the 17 critical synapses led to an average network betweenness *X_B_*(*i,j*) decrease (*V_B_*(*i*,*j*) had positive values) when removed, it suggests that the losses in nodal betweenness centrality *B*(*i*) from synapse loss were greater than any potential nodal betweenness centrality *B*(*i*) increases due to detour path increases. The largest losses appeared to particularly occur when the synapse joined nodes with high nodal betweenness centrality *B*(*i*). Thus, the critical synapses for *V_B_* tended to be those that linked control structures together.

Thus, we again found a strong relationship between betweenness centrality and both *V_E_* and *V_B_*. At the same time, however, there were 5 critical synapses each for *V_E_* (38% of all critical) and *V_B_* (29%) that did not rank as highly for *EBC*: for *V_E_*, synapses 19, 16, 28, 24, and 7 in Table 2; for *V_B_*, synapses 19, 16, 21, 24, and 2 in Table 2) (Tables G and H). These ‘exceptions’ cannot be explained by especially high *Str*, with all critical synapses for *V_E_* between top 19-335 for *Str*, and for *V_B_* between top 35-335 (Tables G and H). Thus, context again appears important. In particular, although the more modest *EBC* values show that these synapses do not have the highest levels of control in the network (regarding number of underlying pathways sharing it), they appear to be especially important for the paths that do traverse the link (compared to the alternative paths).

**Table F.** The Pearson’s correlation coefficient results using all neurons or synapses and correlating the network property measure prior to an attack to the corresponding vulnerability score without using absolute values after an attack

|  | Pearson’s Correlation r (*p* value) | | |
| --- | --- | --- | --- |
|  | *V_C_*(*i*) *without abs* | *V_E_*(*i*) *without abs* | *V_B_*(*i*) *without abs* |
| *Neuron* |  |  |  |
| *D*(*i*) | ***0.510 (0.000)*** | ***0.781 (0.000)*** | ***-0.363 (0.000)*** |
| *Str*(*i*) | ***0.734 (0.000)*** | ***0.909 (0.000)*** | ***-0.374 (0.000)*** |
| *AW*(*i*) | ***0.324 (0.000)*** | ***0.329 (0.000)*** | -0.023 (0.698) |
| *C*(*i*) | ***0.243 (0.000)*** | 0.008 (0.892) | 0.006 (0.919) |
| *E*(*i*) | ***0.275 (0.000)*** | ***0.539 (0.000)*** | -0.107 (0.075) |
| *B*(*i*) | ***0.698 (0.000)*** | ***0.935 (0.000)*** | ***-0.255 (0.000)*** |
| *Synapse* |  |  |  |
| *Str*(*i*,*j*) | ***0.257 (0.000)*** | ***0.468 (0.000)*** | ***0.119 (0.000)*** |
| *EBC*(*i*,*j*) | ***0.117 (0.000)*** | ***0.726 (0.000)*** | ***0.205 (0.000)*** |

**Examination of the relationship between critical neurons and synapses**

The critical synapses were indeed related to the critical neurons, with 27 of the 29 critical synapse neuron pairs (93.1%) containing at least one critical neuron, and 18 of 29 synapses (62.1%) consisting of both critical neurons. To further compare the critical neurons and synapses, we next asked whether the neurons proved critical due to having (a) a critical synapse; (b) multiple critical synapses; or (c) important synapses that are not necessarily critical. Indeed, the critical neurons tended to have multiple critical synapses, with median = 4.5 and range = 0 to 13, although one critical neuron (PVCR) had no critical synapses and two (AVHL and DVA) had only one. At the same time, 10 noncritical neurons had 1 or 2 critical synapses. Thus, although neuron criticality was not necessarily determined by the critical synapses, there was a strong relationship between them.

**Table G.** Network properties of the 29 critical synapses.

| Synapse | | Criticality | | | | Intact network property | | Vulnerability | | |
| --- | --- | --- | --- | --- | --- | --- | --- | --- | --- | --- |
| From | To | *C* | *E* | | *B* | *S*(*i*,*j*) | *EBC*(*i*,*j*) | *V_C_*(*i*,*j*) | *V_E_*(*i*,*j*) | *V_B_*(*i*,*j*) |
| AQR | PVPL |  |  | | 0 | 8 | 0.022 | 0.00159 | 0.00292 | 0.00977 |
| AQR | PVPR |  |  | | 0 | 9 | 0.011 | 0.00094 | 0.00319 | 0.00840 |
| AVAL | AS08 | 0 |  | |  | 9 | 0.002 | 0.03354 | 0.00107 | 0.00060 |
| AVAL | AVAR | 0 |  | |  | 7 | 0.004 | 0.08394 | 0.00032 | 0.00033 |
| AVAL | DA07 | 0 |  | |  | 4 | 0.003 | 0.02130 | 0.00345 | 0.00341 |
| AVAL | PVCL |  | 0 | | 0 | 12 | 0.123 | 0.00683 | 0.00619 | 0.00722 |
| AVAL | VA08 |  | 0 | |  | 19 | 0.010 | 0.00349 | 0.00417 | 0.00179 |
| AVAR | AS08 | 0 |  | |  | 9 | 0.002 | 0.03355 | 0.00052 | 0.00033 |
| AVAR | AVAL | 0 |  | |  | 6 | 0.000 | 0.07595 | 0.00032 | 0.00033 |
| AVAR | DA01 |  | 0 | | 0 | 8 | 0.042 | 0.00574 | 0.00419 | 0.00931 |
| AVAR | DA07 | 0 |  | |  | 3 | 0.000 | 0.02132 | 0.00005 | 0.00021 |
| AVAR | PVCL |  |  | | 0 | 7 | 0.037 | 0.00495 | 0.00148 | 0.01119 |
| AVAR | VA11 |  | 0 | |  | 15 | 0.026 | 0.00363 | 0.00572 | 0.00159 |
| AVEL | AVAL |  | 0 | |  | 12 | 0.061 | 0.00353 | 0.00624 | 0.00039 |
| AVER | AVAR |  | 0 | | 0 | 16 | 0.083 | 0.00407 | 0.00588 | 0.00812 |
| DA01 | AVAR |  | 0 | | 0 | 6 | 0.004 | 0.00391 | 0.00419 | 0.00931 |
| DA01 | VD01 |  |  | | 0 | 17 | 0.048 | 0.00008 | 0.00399 | 0.01258 |
| DVC | PVPR |  | 0 | | 0 | 13 | 0.054 | 0.00104 | 0.00534 | 0.01838 |
| DVC | VD01 |  | 0 | | 0 | 5 | 0.001 | 0.00013 | 0.00633 | 0.01386 |
| PVCL | DVA |  | 0 | |  | 5 | 0.099 | 0.00249 | 0.00981 | 0.00549 |
| PVPL | AQR |  |  | | 0 | 8 | 0.009 | 0.00159 | 0.00292 | 0.00977 |
| PVPR | AQR |  |  | | 0 | 11 | 0.024 | 0.00111 | 0.00321 | 0.00831 |
| PVPR | AVHL |  |  | | 0 | 3 | 0.032 | 0.00003 | 0.00165 | 0.01118 |
| PVPR | DVC |  | 0 | | 0 | 15 | 0.010 | 0.00125 | 0.00534 | 0.01838 |
| RIBL | AVER |  |  | | 0 | 5 | 0.029 | 0.00052 | 0.00222 | 0.00678 |
| RMDL | OLLR |  |  | | 0 | 2 | 0.028 | 0.00007 | 0.00223 | 0.00813 |
| VA08 | DD04 | 0 |  | |  | 21 | 0.008 | 0.01770 | 0.00305 | 0.00000 |
| VA11 | AVAR |  | 0 | |  | 7 | 0.006 | 0.00182 | 0.00440 | 0.00215 |
| VD01 | DVC |  | 0 | | 0 | 5 | 0.074 | 0.00013 | 0.00633 | 0.01386 |
| For all 2990 synapses | | | | Mean | | 2.732 | 0.001 | 0.00084 | 0.00025 | 0.00035 |
|  |  |  |  | SD | | 3.218 | 0.005 | 0.00260 | 0.00064 | 0.00103 |

**Table H.** Rank of network properties of the 29 critical synapses.

| Synapse | | Criticality | | | Intact network property | | Vulnerability | | |
| --- | --- | --- | --- | --- | --- | --- | --- | --- | --- |
| From | To | *C* | *E* | *B* | *S*(*i*,*j*) | *EBC*(*i*,*j*) | *V_C_*(*i*,*j*) | *V_E_*(*i*,*j*) | *V_B_*(*i*,*j*) |
| AQR | PVPL |  |  | 0 | 8 | 160 | 20 | 337 | 43 |
| AQR | PVPR |  |  | 0 | 9 | 130 | 63 | 658 | 40 |
| AVAL | AS08 | 0 |  |  | 9 | 130 | 611 | 4 | 189 |
| AVAL | AVAR | 0 |  |  | 7 | 194 | 200 | 1 | 544 |
| AVAL | DA07 | 0 |  |  | 4 | 461 | 362 | 6 | 28 |
| AVAL | PVCL |  | 0 | 0 | 12 | 69 | 1 | 27 | 5 |
| AVAL | VA08 |  | 0 |  | 19 | 19 | 79 | 106 | 13 |
| AVAR | AS08 | 0 |  |  | 9 | 130 | 603 | 3 | 397 |
| AVAR | AVAL | 0 |  |  | 6 | 261 | 1969 | 2 | 544 |
| AVAR | DA01 |  | 0 | 0 | 8 | 160 | 8 | 45 | 11 |
| AVAR | DA07 | 0 |  |  | 3 | 654 | 1121 | 5 | 1198 |
| AVAR | PVCL |  |  | 0 | 7 | 194 | 9 | 55 | 122 |
| AVAR | VA11 |  | 0 |  | 15 | 35 | 15 | 97 | 7 |
| AVEL | AVAL |  | 0 |  | 12 | 69 | 5 | 101 | 4 |
| AVER | AVAR |  | 0 | 0 | 16 | 27 | 3 | 80 | 6 |
| DA01 | AVAR |  | 0 | 0 | 6 | 261 | 208 | 84 | 11 |
| DA01 | VD01 |  |  | 0 | 17 | 22 | 7 | 2587 | 14 |
| DVC | PVPR |  | 0 | 0 | 13 | 51 | 6 | 588 | 8 |
| DVC | VD01 |  | 0 | 0 | 5 | 335 | 999 | 2379 | 2 |
| PVCL | DVA |  | 0 |  | 5 | 335 | 2 | 171 | 1 |
| PVPL | AQR |  |  | 0 | 8 | 160 | 88 | 337 | 43 |
| PVPR | AQR |  |  | 0 | 11 | 82 | 16 | 542 | 37 |
| PVPR | AVHL |  |  | 0 | 3 | 654 | 11 | 2806 | 109 |
| PVPR | DVC |  | 0 | 0 | 15 | 35 | 80 | 464 | 8 |
| RIBL | AVER |  |  | 0 | 5 | 335 | 12 | 1184 | 69 |
| RMDL | OLLR |  |  | 0 | 2 | 920 | 13 | 2615 | 68 |
| VA08 | DD04 | 0 |  |  | 21 | 13 | 98 | 7 | 42 |
| VA11 | AVAR |  | 0 |  | 7 | 194 | 137 | 276 | 10 |
| VD01 | DVC |  | 0 | 0 | 5 | 335 | 4 | 2379 | 2 |

**References**

1. Barrat A, Barthelemy M, Pastor-Satorras R, Vespignani A. The architecture of complex weighted networks. Proceedings of the National Academy of Sciences of the United States of America. 2004;101(11):3747.

2. Newman MEJ. Analysis of weighted networks. Physical Review E. 2004;70(5):056131.

3. Rubinov M, Sporns O. Complex network measures of brain connectivity: uses and interpretations. Neuroimage. 2010;52(3):1059-69.

4. Opsahl T, Agneessens F, Skvoretz J. Node centrality in weighted networks: Generalizing degree and shortest paths. Social Networks. 2010;32(3):245-51.

5. Krzywinski M, Schein J, Birol I, Connors J, Gascoyne R, Horsman D, et al. Circos: an information aesthetic for comparative genomics. Genome research. 2009;19(9):1639-45.

6. Fagiolo G. Clustering in complex directed networks. Physical Review E. 2007;76(2):026107.

7. Latora V, Marchiori M. Efficient behavior of small-world networks. Physical Review Letters. 2001;87(19):198701.

8. Freeman LC. Centrality in social networks conceptual clarification. Social Networks. 1979;1(3):215-39.

9. Girvan M, Newman ME. Community structure in social and biological networks. Proceedings of the national academy of sciences. 2002;99(12):7821-6.

10. Varshney LR, Chen BL, Paniagua E, Hall DH, Chklovskii DB. Structural properties of the *Caenorhabditis elegans* neuronal network. PLoS computational biology. 2011;7(2):e1001066.

11. Piggott BJ, Liu J, Feng Z, Wescott SA, Xu X. The Neural Circuits and Synaptic Mechanisms Underlying Motor Initiation in *C. elegans*. Cell. 2011;147(4):922-33.

12. Altun Z, Hall D. WormAtlas. Available at http://www.wormatlas.org. 2002.

13. Sawin ER, Ranganathan R, Horvitz HR. *C. elegans* locomotory rate is modulated by the environment through a dopaminergic pathway and by experience through a serotonergic pathway. Neuron. 2000;26(3):619-31.

14. Wicks SR, Roehrig CJ, Rankin CH. A dynamic network simulation of the nematode tap withdrawal circuit: predictions concerning synaptic function using behavioral criteria. The Journal of neuroscience. 1996;16(12):4017-31.

15. Inglis PN, Ou G, Leroux MR, Scholey JM. The sensory cilia of *Caenorhabditis elegans*. WormBook: The Online Review of C elegans Biology. 2005.

16. White JG, Southgate E, Thomson J, Brenner S. The structure of the nervous system of the nematode *Caenorhabditis elegans*. Philosophical Transactions of the Royal Society of London B, Biological Sciences. 1986;314(1165):1-340.

17. Ardiel EL, Rankin CH. Cross-referencing online activity with the connectome to identify a neglected but well-connected neuron. Current Biology. 2015;25(10):R405-R6.

18. Hilliard MA, Bargmann CI, Bazzicalupo P. *C. elegans* responds to chemical repellents by integrating sensory inputs from the head and the tail. Current Biology. 2002;12(9):730-4.

19. Durbin RM. Studies on the development and organisation of the nervous system of *Caenorhabditis elegans*: University of Cambridge; England; 1987.

20. Chao MY, Larkins-Ford J, Tucey TM, Hart AC. *lin-12* Notch functions in the adult nervous system of C. elegans. BMC neuroscience. 2005;6(1):45.

21. Freeman LC. A set of measures of centrality based on betweenness. Sociometry. 1977:35-41.

22. Kaiser M, Hilgetag CC. Edge vulnerability in neural and metabolic networks. Biological cybernetics. 2004;90(5):311-7.
