# Supplementary figures and images for: Vulnerability-Based Critical Neurons, Synapses, and Pathways in the Caenorhabditis elegans Connectome
Source: PLoS Comput Biol. 2016 Aug 19;12(8):e1005084. doi: 10.1371/journal.pcbi.1005084 (PMC4991803; doi:10.1371/journal.pcbi.1005084)

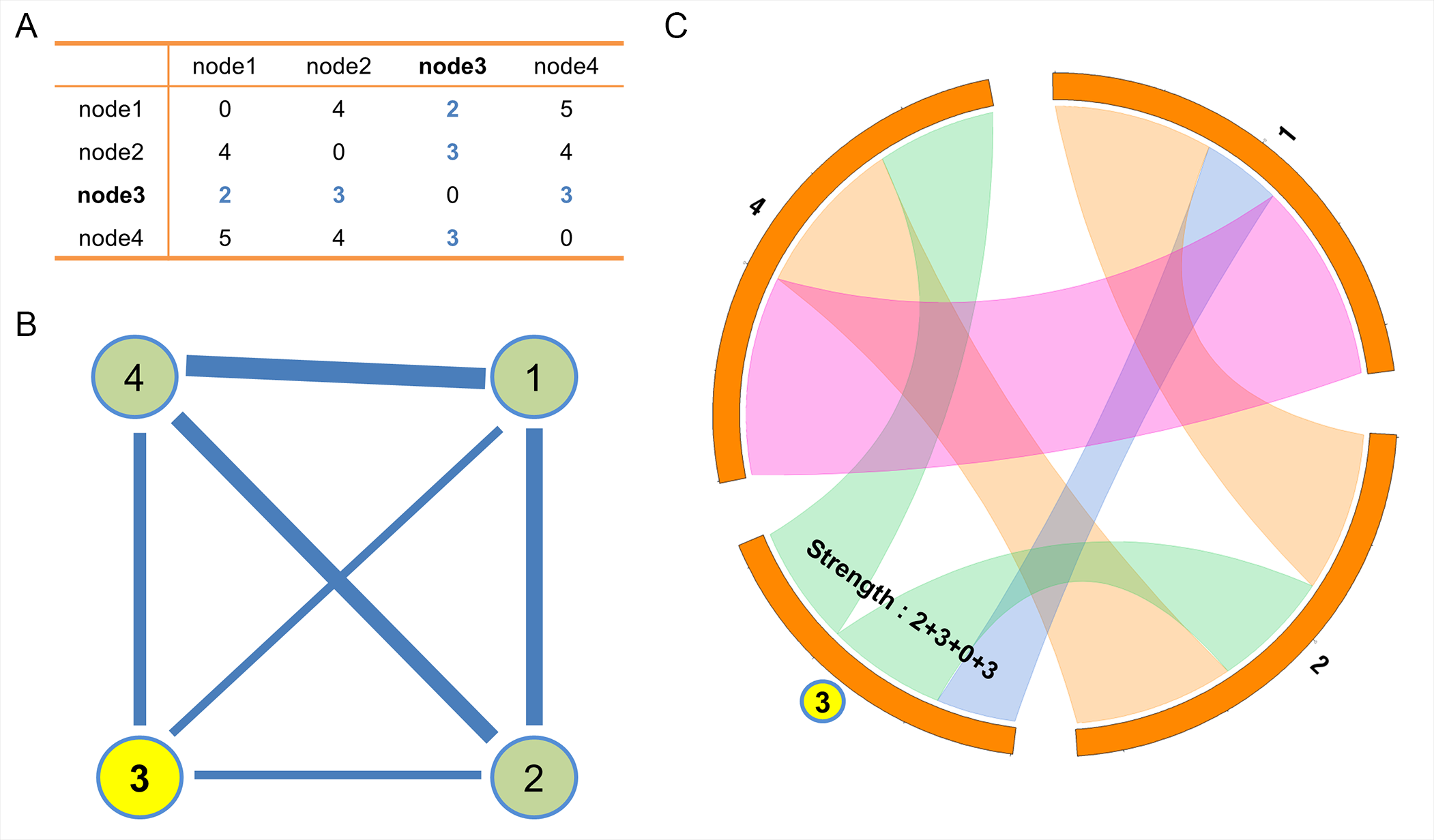

Supplement: S1 Fig — (A) Adjacency matrix of an example network. (B) Schematic graph drawing of the example network. (C) Undirected circular wiring diagram. (TIF) [file pcbi.1005084.s002.tif]

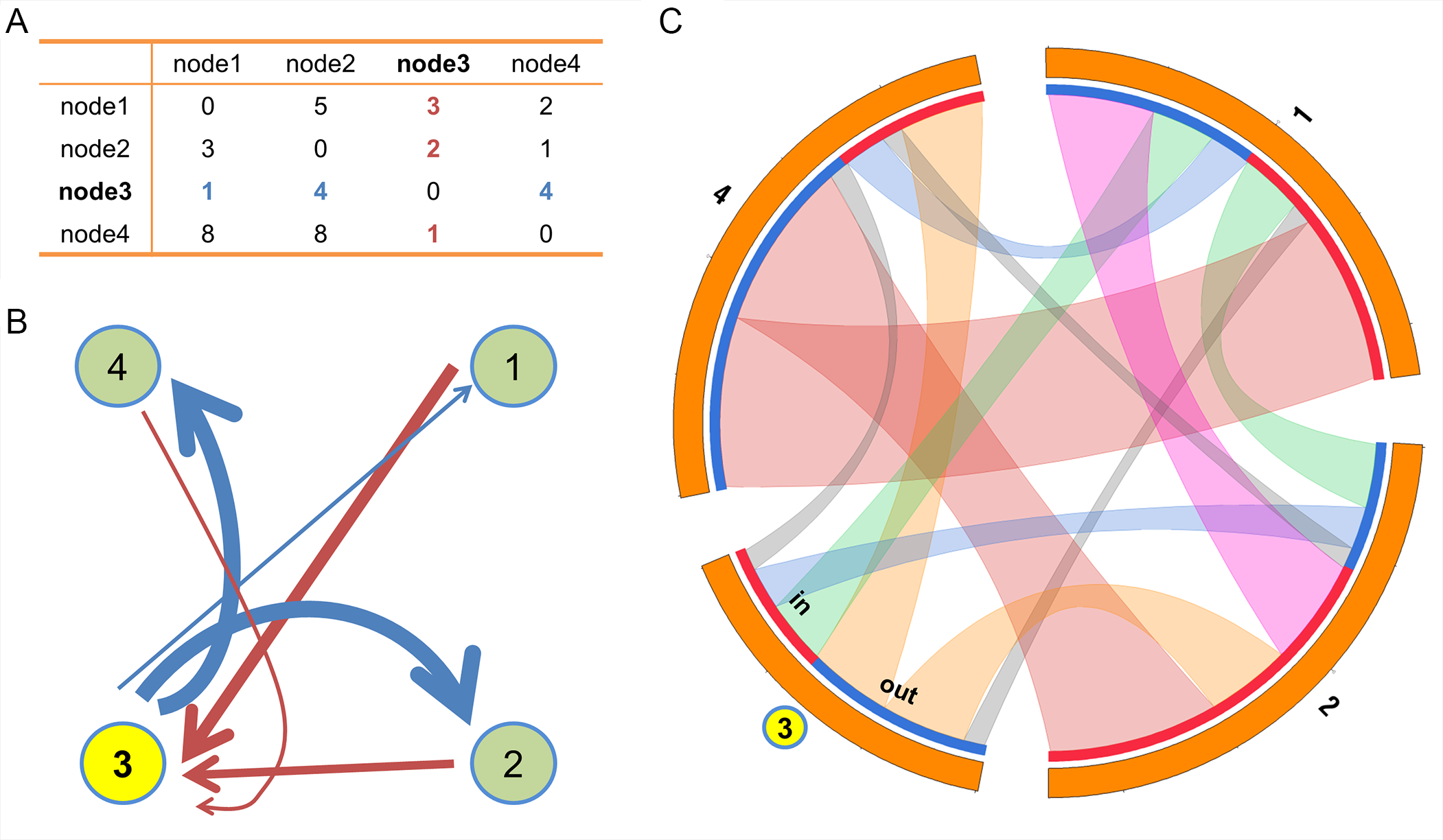

Supplement: S2 Fig — (A) Adjacency matrix of an example network. (B) Schematic graph drawing of the example network focused on the connections of the node 3. (C) Directed circular wiring diagram. (TIF) [file pcbi.1005084.s003.tif]

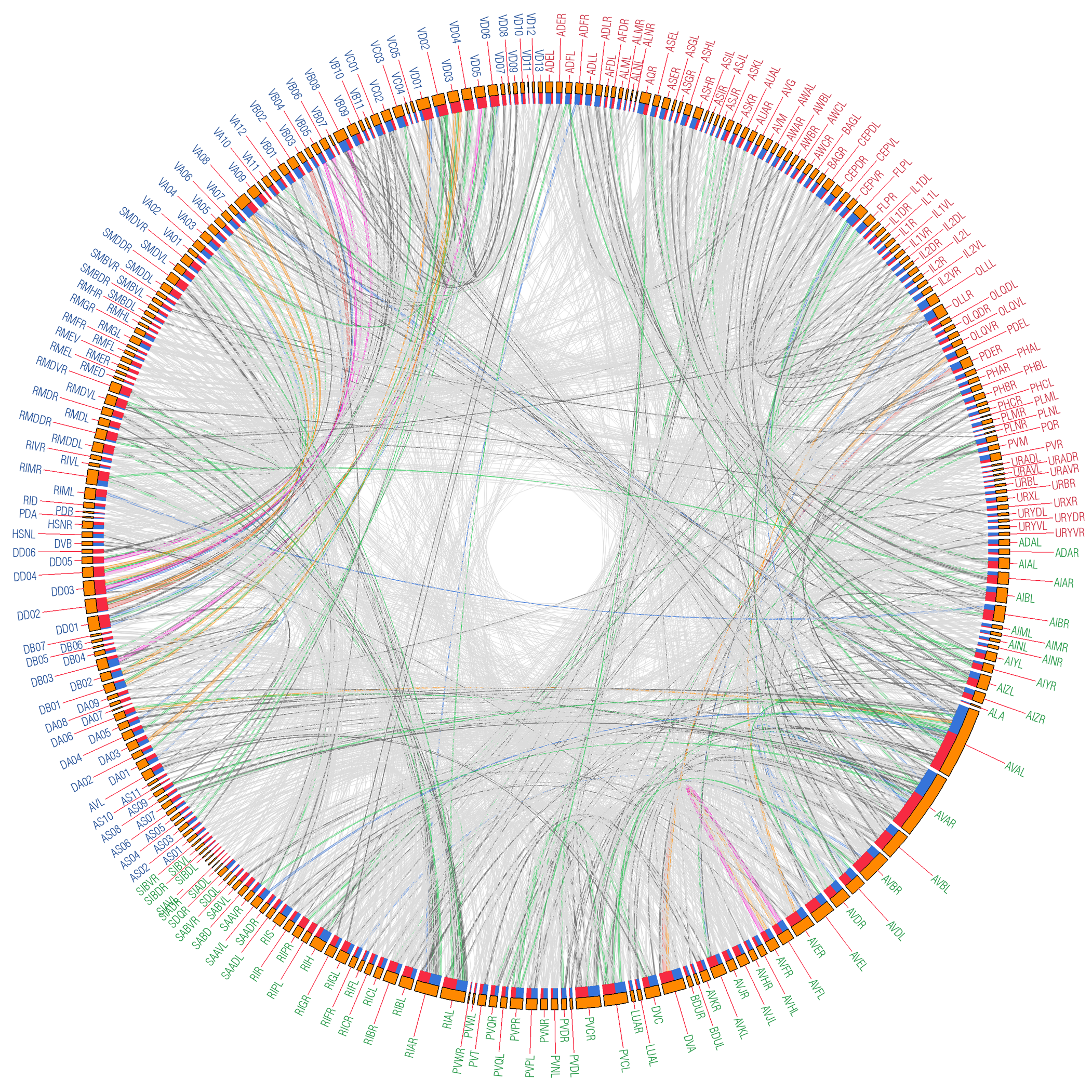

Supplement: S3 Fig — Link colors show the weights of each connection (light grey: 1–5, grey: 6–10, green: 11–15, blue: 16–20, orange: 21–25, pink: 26–30, and red: over 30). The colors of the names of neurons indicate their neuronal types (sensory neuron: red; interneuron: green; and motor neuron: blue). The lengths of the segments of the outer layer (orange) indicate the overall strength (Str) of the nodes. The lengths of the red segments (sinks) indicate the total Strin of the nodes, and the lengths of the blue segments (sources) indicate the total Strout of the nodes. (TIF) [file pcbi.1005084.s004.tif]

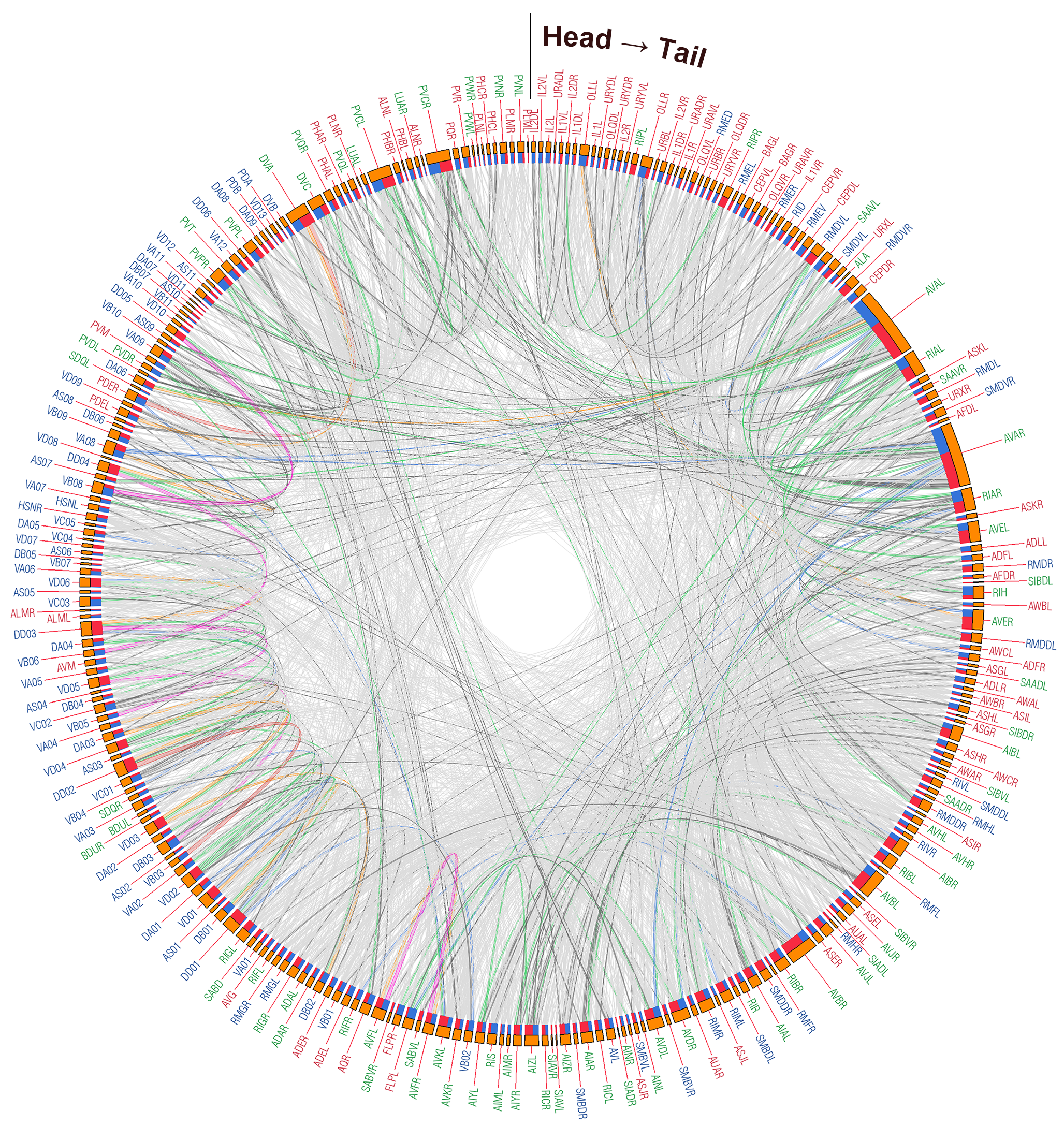

Supplement: S4 Fig — Link colors show the weights of each connection (light grey: 1–5, grey: 6–10, green: 11–15, blue: 16–20, orange: 21–25, pink: 26–30, and red: over 30). The colors of the names of neurons indicate their neuronal types (sensory neuron: red; interneuron: green; and motor neuron: blue). The lengths of the segments of the outer layer (orange) indicate the strength (Str) of the nodes. The lengths of the red segments (sinks) indicate the total Strin of the nodes, and the lengths of the blue segments (sources) indicate the total Strout of the nodes. (TIF) [file pcbi.1005084.s005.tif]

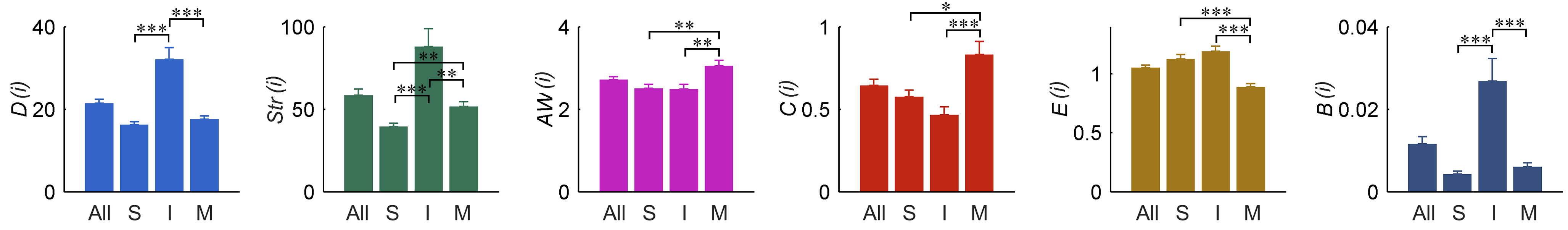

Supplement: S5 Fig — Bar plots indicate the results of each measure by neuronal types (All: entire 279 neurons; S: 88 sensory neurons; I: 82 interneurons; and M: 109 motor neurons). Error bars represent standard error of the mean. *: P < 0.05, **: P < 0.01, ***: P < 0.001. Interneurons had higher values of degree (D, i.e., the number of connections), strength (Str, i.e., the number of connections times their weight), nodal efficiency (E(i), i.e., the average shortest path between the neuron and all others), and nodal betweenness centrality (B(i), i.e., a measure of the degree to which shortest paths travel through the unit) than the other neuronal types. Motor neurons had higher average weight (AW, i.e., the average strength of connections) and nodal clustering coefficient (C(i), i.e., the degree to which its neighbors are connected with each other) values and lower E values than the other neuronal types. Sensory neurons had generally lower Str values than the other two. (TIF) [file pcbi.1005084.s006.tif]

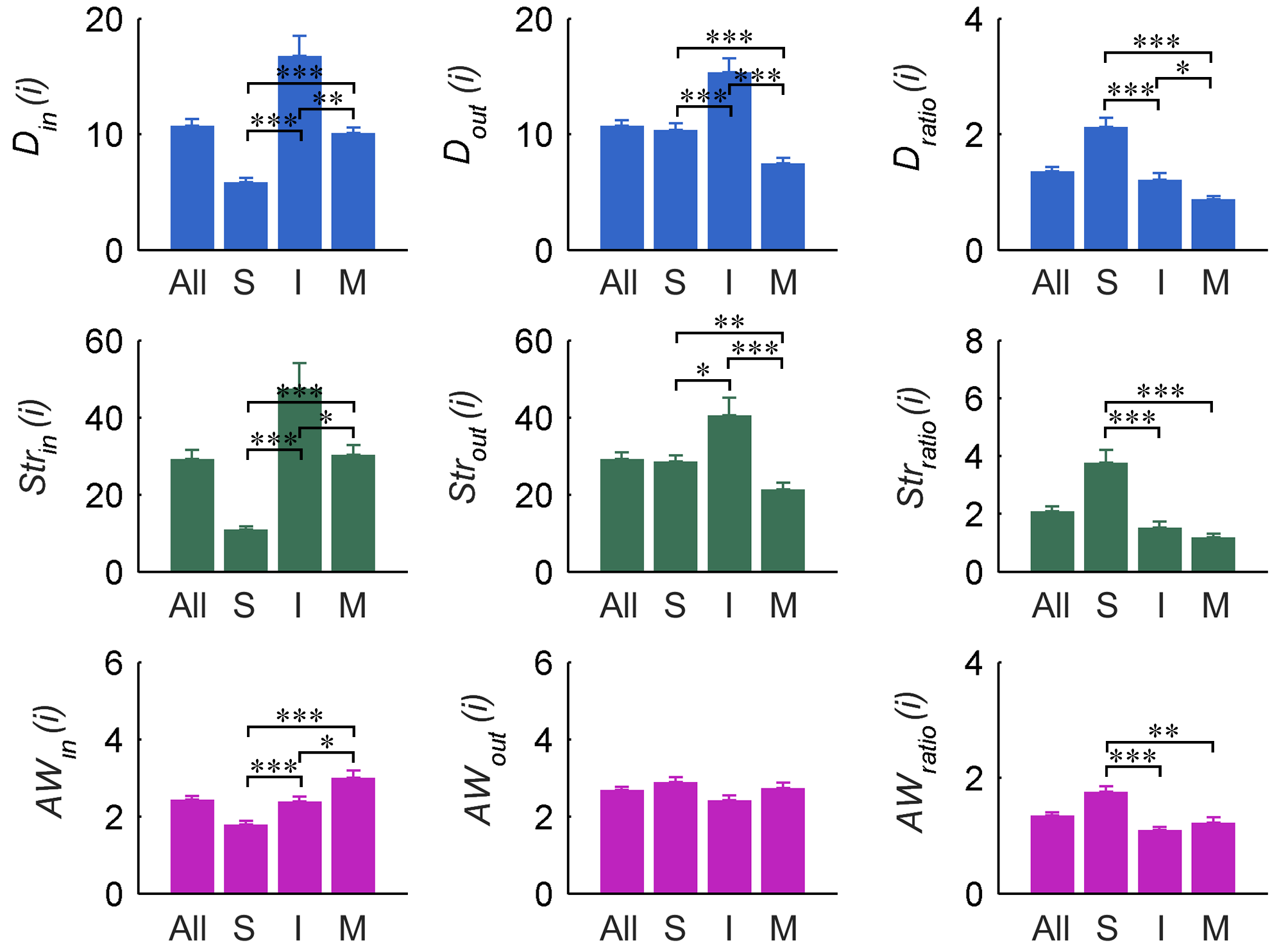

Supplement: S6 Fig — Directional information is indicated as input (in) and output (out). The ratio of the out to the in value (ratio) is also plotted. Bar plots indicate the results of each measure by neuronal types (All: entire 279 neurons; S: 88 sensory neurons; I: 82 interneurons; and M: 109 motor neurons). Error bars represent standard error of the mean. *: P < 0.05, **: P < 0.01, ***: P < 0.001. The results for both degree and strength (i.e., all connection weights summed) are the same, with interneurons having significantly higher in and out directions (for both D and Str) than the other types, motor neurons having significantly higher in direction than sensory neurons, and sensory neurons having significantly higher out direction than motor neurons. Additionally, sensory neurons have a significantly higher Dratio (ratio of out to in) than the other types, while interneurons also have a significantly higher Dratio than motor neurons. At the same time, motor neurons have significantly higher AWin than the other types, and interneurons have higher AWin than sensory neurons (with no significant differences in AWout). Sensory neurons also have a significantly higher AWratio than the other types. (TIF) [file pcbi.1005084.s007.tif]

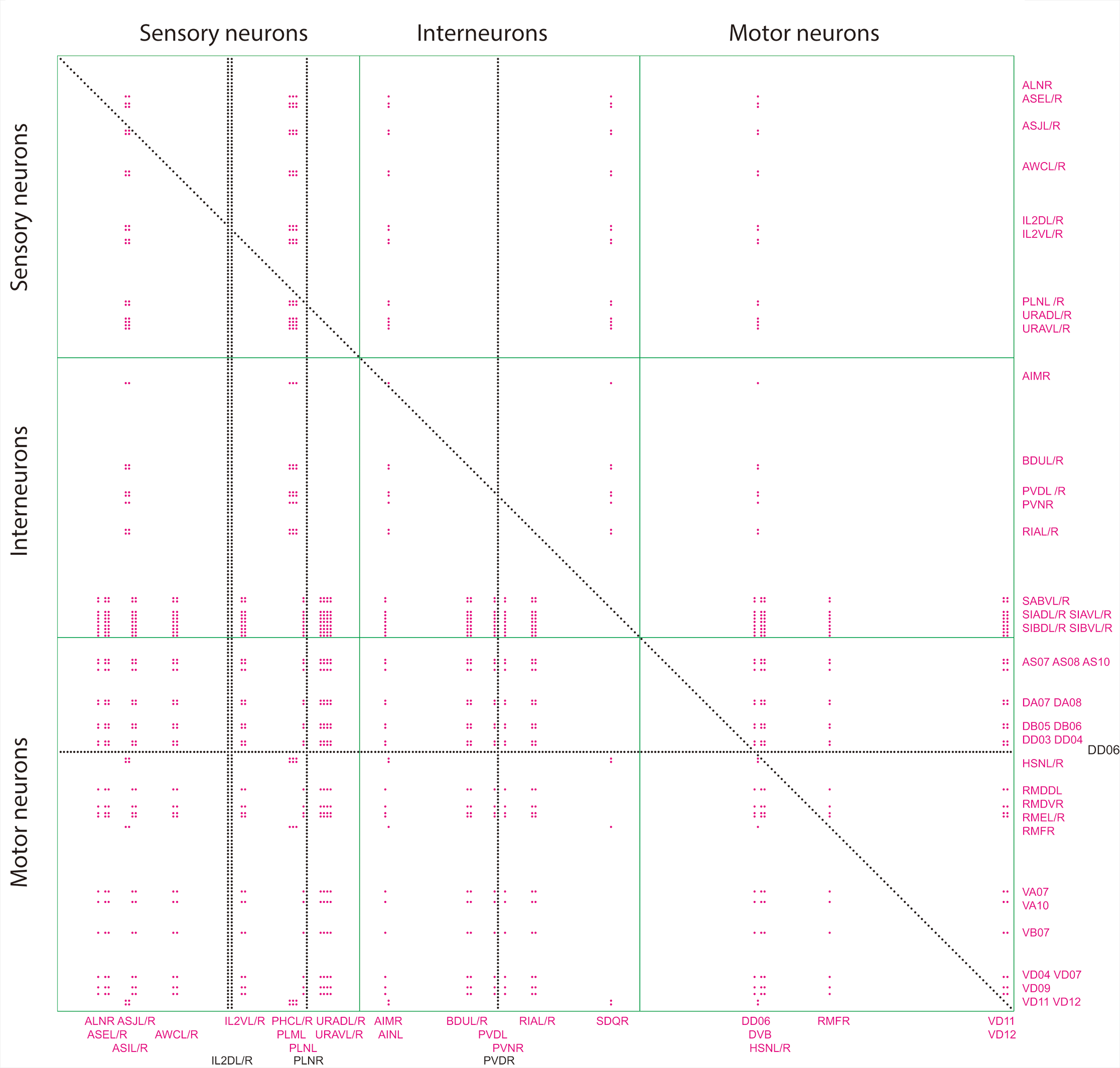

Supplement: S7 Fig — The color of an element aij depicts the possibility of reachability by network types: i.e., gap junction, chemical synapse, and full networks (pink: the full network only, black: impossible reachability, and white: possible reachability for all networks). (TIF) [file pcbi.1005084.s008.tif]

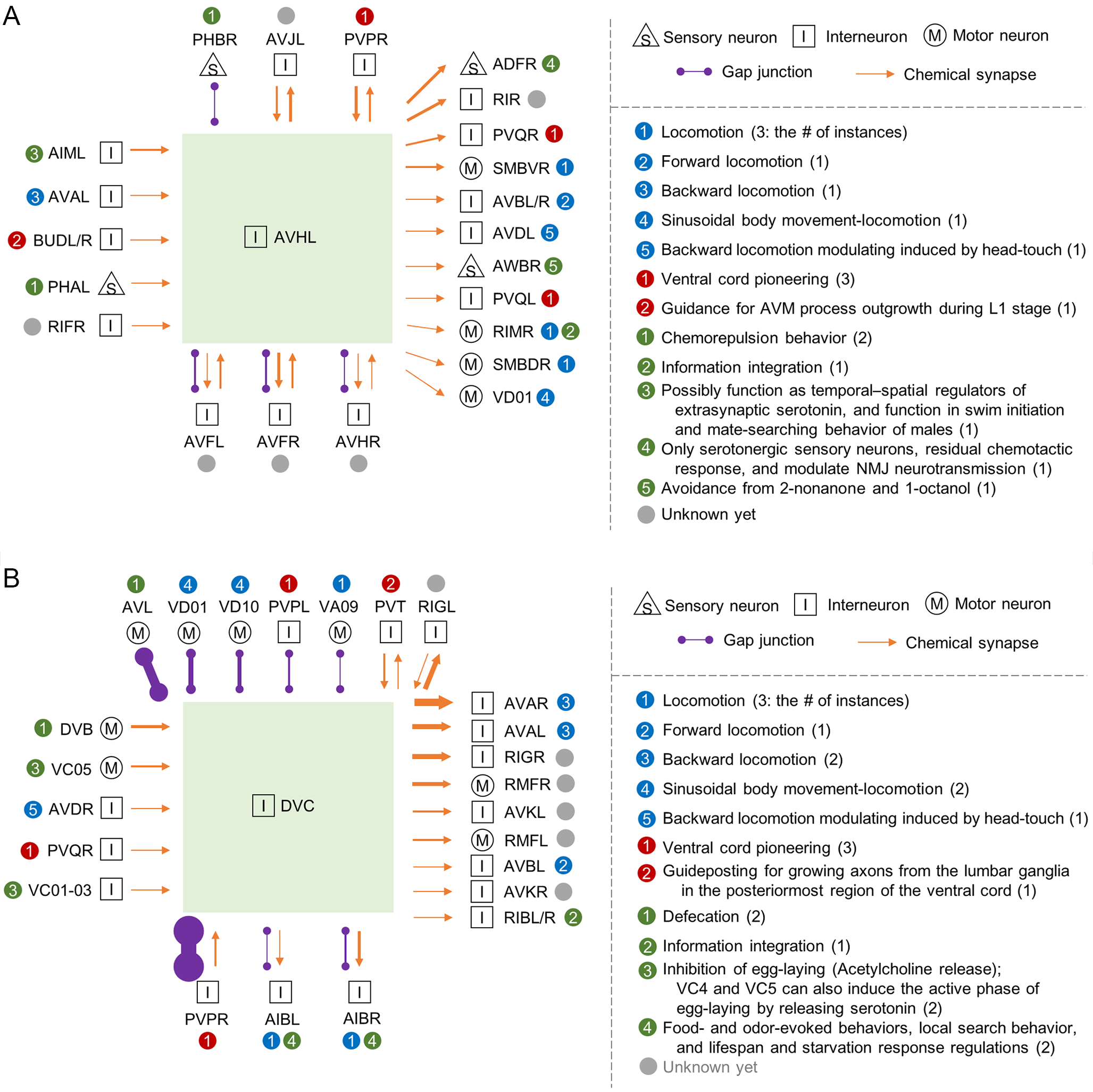

Supplement: S8 Fig — (A) AVHL and (B) DVC. Biological functions were divided into four categories: (1) blue: body movement or locomotion; (2) red: pioneering, growth, and neuronal development; (3) green: chemical reactions or information integration; (4) gray: unknown function. The number in the parentheses is the count of instances for each specific biological function. Synapse types are also denoted as purple for gap junction and orange for chemical synapse. (TIF) [file pcbi.1005084.s009.tif]
